# Characterizing preferred motif choices and distance impacts

**DOI:** 10.1371/journal.pone.0215242

**Published:** 2019-04-16

**Authors:** Jinzhou Cao, Qingquan Li, Wei Tu, Feilong Wang

**Affiliations:** 1 State Key Laboratory of Information Engineering in Surveying, Mapping and Remote Sensing, Wuhan University, Wuhan, P.R. China; 2 Shenzhen Key Laboratory of Spatial Information Smart Sensing and Services and Research Institute for Smart Cities, Department of Urban Informatics, School of Architecture and Urban Planning, Shenzhen University, Shenzhen, P.R. China; 3 Key Laboratory for Geo-Environmental Monitoring of Coastal Zone of the National Administration of Surveying, Mapping and GeoInformation, Shenzhen University, Shenzhen, P.R. China; 4 Department of Civil and Environmental Engineering, University of Washington, Seattle, Washington, United States of America; Central South University, CHINA

## Abstract

People’s daily travels are structured and can be expressed as networks. Few studies explore how people organize their daily travels and which behavioral principles result in the choices of specific network types. In this study, we first reconstruct location networks and activity networks for numerous individuals from high-resolution mobile phone positioning data and define frequent networks as motifs. The results suggest that 99.9% of people’s travels can be characterized by a limited set of location-based motifs and activity-based motifs. The results further reveal that the least effort principle governs the preferred motif choices through quantifying the rank-frequency properties. The scaling properties of distance characteristically impact motifs, and their scaling differences by node numbers and motif types coincide with the popularities of motifs, verifying the self-adaptions in motif choices; that is, although individuals travel with unique propensities, they always tend to choose the motif with the lowest consumption that satisfies their demand.

## Introduction

Uncovering hidden patterns and statistical properties of human mobility is currently one of the most dominant topics in the field of statistical physics, geography, transportation, and urban planning[1–5]. Human mobility has been empirically observed to exhibit a high degree of spatial-temporal regularity[6, 7]. It is further reported that human travelers are not random walkers when exploring the physical space[8]. However, few studies have explored the network structure of the daily travel of humans. People always plan their daily travel in terms of destination, duration, and travel route. Their itineraries can be modeled as daily travel motifs, a set of subgraphs representing a universal class of networks. The daily travel motifs are analogous to the concept of motifs from the complex network theory, which has been widely applied to biological or ecological networks[9]. Schneider et al. [10] brought this concept to human mobility studies. In the scenarios of human travel, motifs are defined as frequent occurred networks, where visited locations and trips are detected. Motifs abstracted from heterogeneous human travel are structured, making it easy to understand universal mobility patterns. Understanding motifs behind daily human travel benefits further investigations on how people organize and determine their motif structures and underlying mechanisms of the behaviors of motif choices[11].

Statistical metrics can be developed to provide useful insights into the popularity of motifs. For instance, the travel distance embedded in a motif is viewed as the integration of multiple factors that people must consider when choosing a specific motif, such as mobility regularities, travel costs and spatial boundaries[12,13]. Hence, investigation of the travel distance provides another perspective on understanding motif choice behaviors. The importance of distance can be explored by uncovering its scaling characteristics. The probability distribution of travel distance is empirically observed to follow disparate functional forms in multiple proxy data sources and scales, such as the power-law[6, 7, 14–18], log-normal[17, 19, 20] and exponential distributions[21–23]. Furthermore, certain studies attempt to explain the driving forces underlying these distributions. These driving forces are highlighted by animal foraging behaviors (random walks)[24], banknote circulations[14], exploratory and preferential returns[6], hierarchical organizations of traffic systems[25], and combinations of transportation modes[17].

The development of information and communication technology (ICT) advances the magnanimous yet heterogeneous mobility data to characterize human mobility, such as call detail records (CDRs)[26,27], mobile phone positioning data[28,29], GPS trajectories[30,31], and social media data[21,32], which have various advantages because of the unparalleled scales and high resolutions[33]. Although these data-driven studies have resulted in significant findings, they still face great challenges. First, current research always expresses human travel as a trajectory. However, it is difficult to obtain uniform measurements when modeling travel pattern at the trajectory level. A structured representation of the trajectory is needed. While existing studies constructing motifs using CDR data and survey data as their proxies of human travel suffer from sparsity in space and time, thus generating natural drawbacks in constructing complete motif structures. In addition, motifs are only generated from the location perspective thus are lack of the activity perspective. Second, although certain studies have focused on the exploration of the driving forces of human travel, there is a lack of knowledge of behavioral principles on how people choose their travel networks. Third, in trying to understand aggregated mobility, statistics using different aggregations may address differences in descriptive conclusions. Therefore, the influences caused by individual heterogeneities cannot be neglected.

In this study, we reconstruct individual mobility motifs and then uncover hidden patterns, which deepen the understanding of motif choices by using a high-resolution mobile phone positioning dataset of 9.7 million users: 1) by characterizing the motif choices with rank-frequency distributions, we revealed the general mechanism of motif choice behaviors; 2) by investigating the average travel distance in the motifs, we determined the best-fitted probability distribution function (PDF) to reveal the scaling properties and to explain the relationship between the physical significance of the parameters and mobility mechanisms; and 3) by investigating the distance scaling properties conditional on motif heterogeneities, we noted the distance scalings are correlated with the popularities of motifs. We verified that the least effort principle governs the motif choices and the travel distance did impacts on motif choices and the scaling differences revealed the travel self-adaptions in motif choices. The depiction of travel motif choices and their distance scaling properties can refine our understanding of human mobility and benefit the elaboration of urban planning[34,35], traffic optimization[36,37], disease spreading[38,39], and so on. The main contributions of this study are as follows: 1) uncovering the location-based and activity-based motifs behind massive daily travel from raw mobile phone positioning data. The scaling laws of distance characteristically impact motifs, and their scaling differences by node numbers and motif types suggest travel self-adaptions in motif choices; 2) revealing the mechanism of motif choices. The least effort principle has been observed to drive human travel through qualifying of the properties of motif choices; 3) instead of using intrinsically deficient or small-size sample data, a set of reliable mobile phone positioning data were used to abstract individuals’ trajectories.

## Methods

### Data

**Fig 1:**
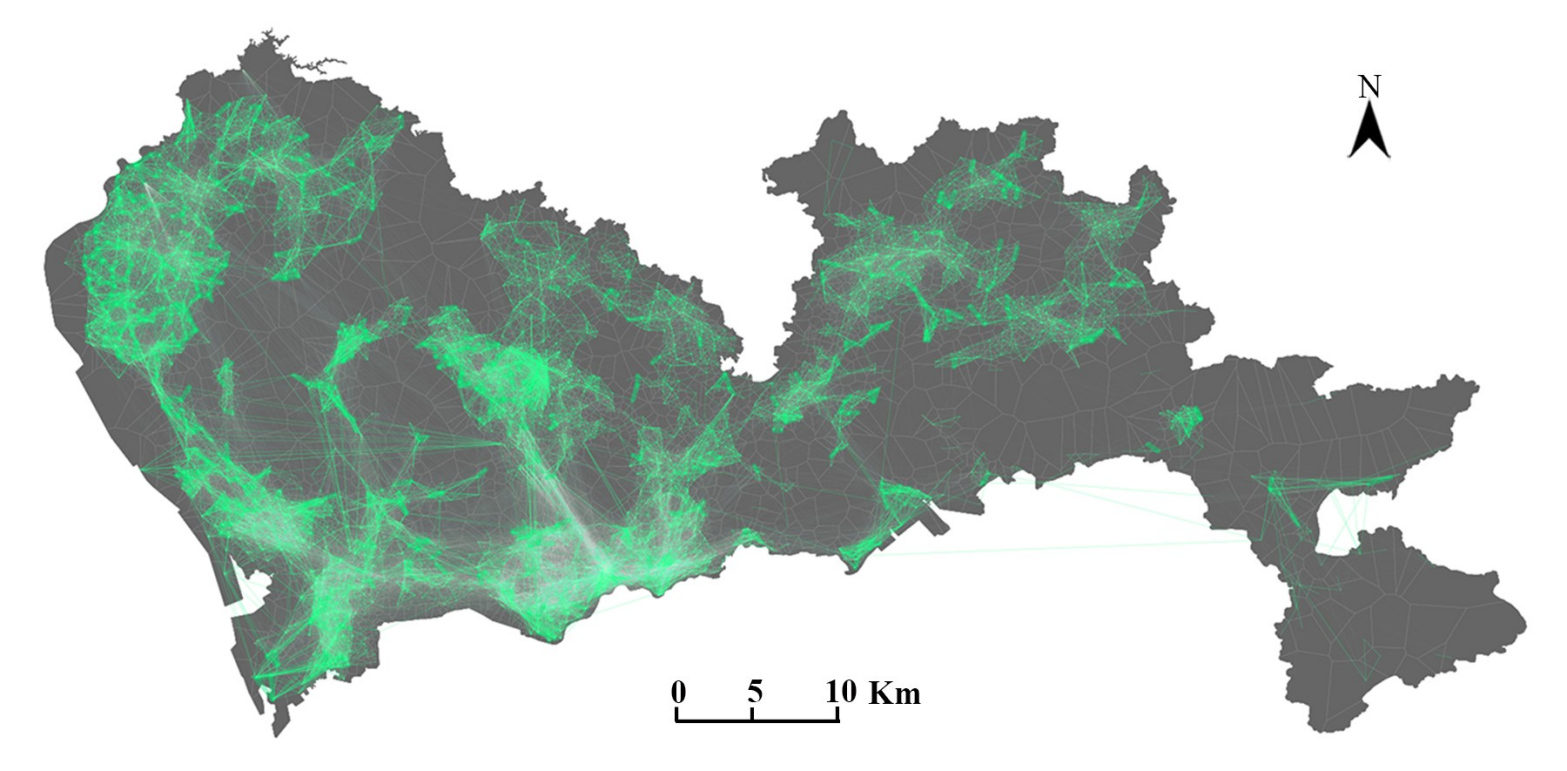
The study area in Shenzhen, China, and travel flows extracted from the mobile phone positioning data at the base tower level. There are 5,929 cell phone towers, and the polygons were approximated by Voronoi tessellation of the towers representing the corresponding service areas. This dataset contains the positioning data of 9.7 million phone users (approximately 57.5% of the total population) during a workday in March 2012. The thicker lines indicate that more travel flows occurred between the two Voronoi polygons. The figure was created with an open source visualization toolkit: Processing (https://processing.org/). The administrative division of a shapefile sourced from the Bureau of Planning and Natural Resources of Shenzhen (http://pnr.sz.gov.cn/ywzy/chgl/bzdtfw/).

The mobile phone positioning data were provided by a major communication in Shenzhen, China. The dataset was recorded in a workday in March 2012, as visualized in Fig 1. The positions of users had been recorded at hourly intervals at the base tower level; thus, each user has at least 24 records including the user id, time-stamp, and latitude and longitude of the base towers. After removing duplicates, 332,624,029 observations remained. This dataset comprised 9,702,082 users, which was approximately 57.5% of the total population in Shenzhen City. This result indicates the advantageous penetration rates compared to CDR records or other traditional travel survey datasets. To protect the phone user privacy, the dataset had been anonymized by the communication company. Any personal information, such as phone number, user name, gender, and age, cannot be accessed in the data processing.

### Construction of the motifs

**Fig 2:**
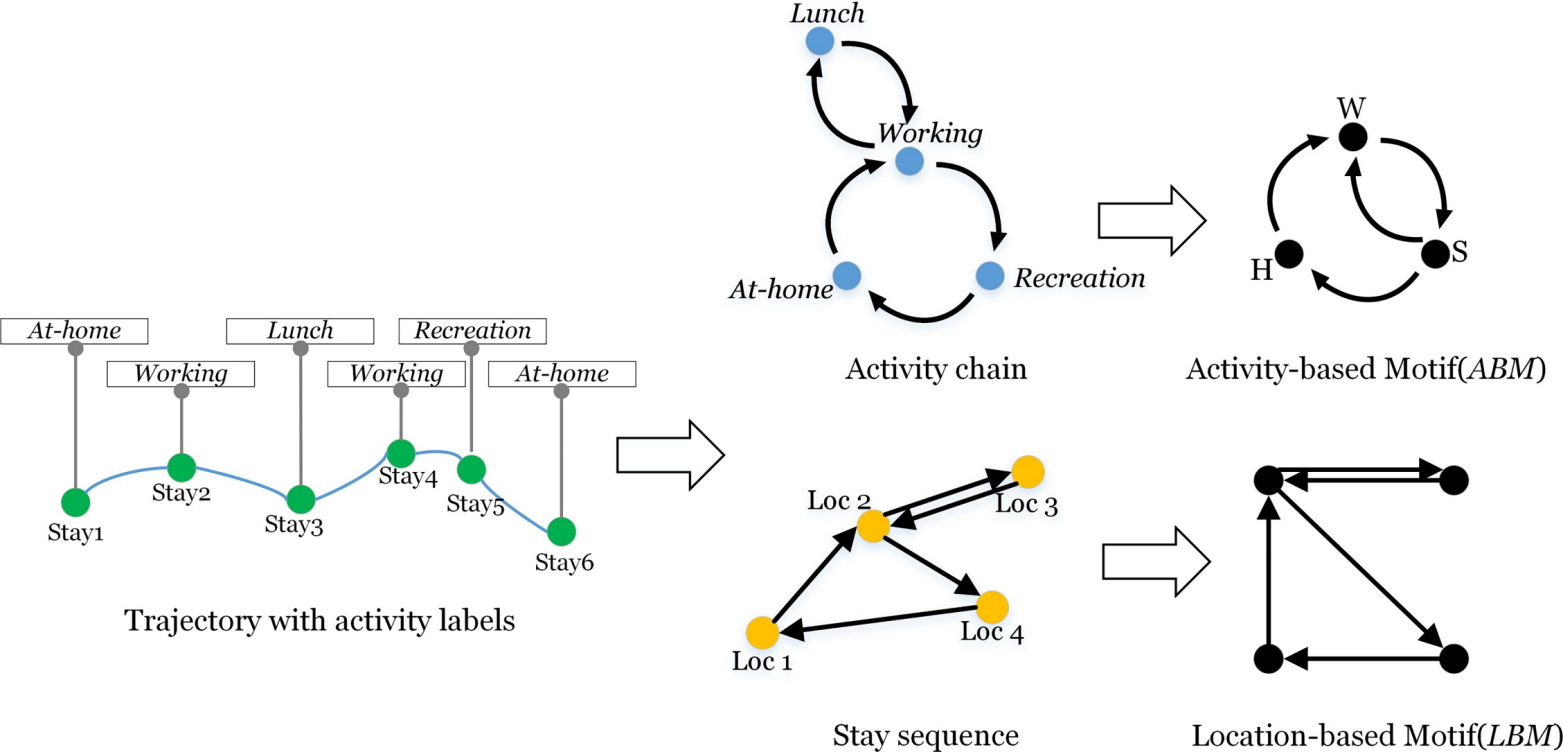
The illustration of location-based and activity-based motifs constructed from stay sequences and activity chains.

The individual trajectory was abstracted as a motif from raw mobile phone positioning data by using a three-step method. As illustrated in Fig 2, the raw data was firstly segmented into the stay sequences. Then the activity labels, such as in-home, working, social activities were annotated to each stay. Finally, the stay sequence with activity labels was used to extract two types of directed weighted networks: a **location-based motif** (***LBM***) and an **activity-based motif (*ABM*)** person by person.

#### Stay extraction

A sequence of stays representing the locations where users engaged in activities was extracted from time-sequential positioning records[40]. We adopted a tower-based segmentation algorithm by using both spatial and temporal rules. The records were firstly sorted by time. Given the uncertainty of data collection, time-consecutive records satisfying the spatial constraint (500 meters) and temporal constraint (duration of 60 minutes or longer) were clustered as stays. Once a stay was identified, for simplicity, the coordinates of the stay were set as the coordinates of the tower which had the maximum number of records belonging to that stay, as seen in **S1 Text**. Twenty-four records were processed for each person, and thus, sequences of stays for each person were obtained.

#### Home/Work/Social activities detection

According to the circadian rhythms and regularities behind the daily cycles[41], the activity labels of stays were determined. Using time-windows and durations of stays, we detected in-home/working/social activities as follows. (a) If the duration of one stay occupies more than half of the time-window at early morning hours (0:00–6:00), the location of this stay would be defined as home. All activities located at the home location of this user were detected as in-home activities. (b) If the duration of one stay occupies more than half of the time-window at working hours (9:00–12:00 and 14:00–17:00), the location of this stay would be defined as the workplace. All activities located in the workplace of this user were detected as working activities. (c) All stays that are not labeled as the home or working activities were detected as social activities.

#### Motif construction

Let the stay sequence and corresponding activity chain for each user be *S*_*Loc*_ = {*Loc*_1_,*Loc*_2_…,*Loc*_*N*_} and *S*_*Acti*_ = {*Acti*_1_,*Acti*_2_…,*Acti*_*M*_}, *N* is the number of distinct visited locations, and *M* is the number of activity types. The location-network was constructed from a spatial perspective. Thus, the structure of location-network, *V*_*Loc*_ = (*N*,*E*) was constructed from *S*_*Loc*_, where *N* is the nodes equaling the visited places, and *E* is the directed edges between nodes, equaling the trips between locations. The activity-network was extracted from the activity space. Thus, the structure of activity-network, *V*_*Acti*_ = (*M*,*E*) was constructed from *S*_*Acti*_, where *N* is activity types, and *E* is the directed edges between nodes, equaling transitions between activities. Essentially, *V*_*Loc*_ and *V*_*Acti*_ were both expressed in weighted matrix forms. Finally, each user's daily travel was abstracted to a location-network and an activity-network. We identified the frequent networks as **location-based motifs (*LBMs*)** and **activity-based motifs (*ABMs*).** The number of nodes in a *LBM* was abbreviated as the ***LN***, while that in an *ABM* was abbreviated as the ***AN*.** The *LBM* and *ABM* from one individual trajectory exhibited their intertwined relationship. We referred to the correspondent combination of two motif types for each person as the **joint motif** (***JM***). The properties of the constructed motifs are illustrated in **S1 Text.**

### Discrete generalized beta distribution (DGBD)

The *DGBD* is a quantitative model for statistical behaviors expressed by a rank-frequency distribution and has been well studied in social and natural sciences[42]. A *DGBD* system does not show pure Zipf-like behavior in the whole range but exhibits truncated scaling behavior in the tail part. Unlike the Zipf's law with one exponent[43], the *DGBD* introduces a second exponent to control the curvature of the tail part, such that the model can justify the finite-size effect[44]. Therefore, the *DGBD* is expressed by a power-law-like regime for small rank values (frequent occurrences), followed by a truncated regime with steeper decays for large rank values (infrequent occurrences). The *DGBD* outperforms Zipf's law in portraying the scaling behaviors in rank-frequency distributions. It should be noted that Zipf's law is considered a special form of the *DGBD* because the *DGBD* reduces to Zipf's law when *γ* = 0.

### Fitting procedure

To determine which distribution best fits the empirical data and evaluate how well it fits, inspired by the method proposed by Clauset et al.[45], we selected an integrated fitting procedure, called the bootstrap-Kolmogorov-Smirnov test. It should be noted that even though using the regression method on log-log plots to estimate parameters is biased[46,47], many studies still use this method for fitting. (1) Determine *D*_*fit*_*min*_ that minimizes the value of the KS statistic using the Kolmogorov-Smirnov (KS) test; (2) Estimate the parameters *α* and *κ* using the maximum likelihood estimation method (MLE); (3) Calculate the KS statistic D* for the empirical data and the best-fitted model; (4) Generate *n* sets of synthetic data from the best-fitted model; (5) Compute the MLE parameters and estimate the KS statistic for each synthetic data set; obtain the distribution of KS statistics *P*(*D*) of *D*_1_,*D*_2_…,*D*_*n*_; (6) Count the fraction of *P*(*D*) greater than or equal to D*, which indicates the fitness significance level (*p-value*). A *p-value* close to 1 indicates that the empirical data matches its best fit as good as synthetic data, whereas a relatively small *p-value* (typically chose p < 0.10) would suggest that the empirical data cannot be the result of its best fit. We chose *n* = 2500 to guarantee the correctness of goodness of fit following the suggestion of paper[45].

## Results

### Properties of the preferred motif choice

**Fig 3:**
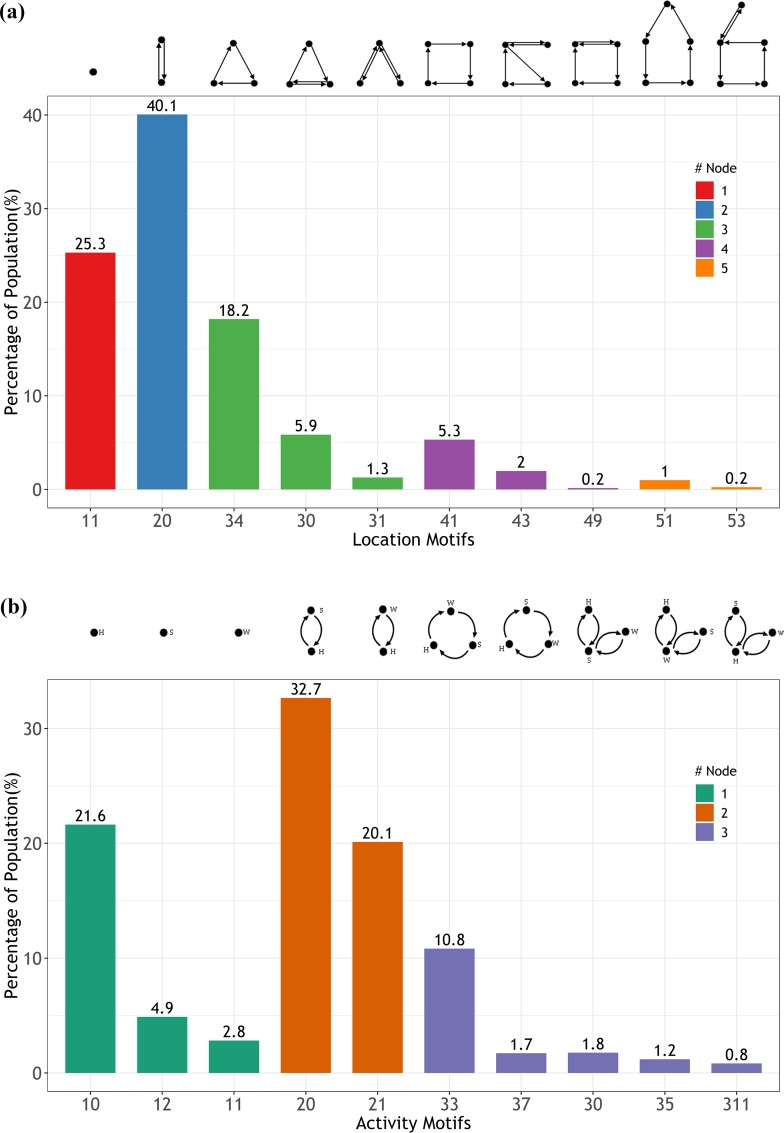
**(a) The probability distribution of location-based motifs (probability > 0.1%). (b) The probability distribution of activity-based motifs (probability > 0.5%).** The different colors indicate the number of component nodes in a motif. The topological network structures are shown at the top.

After processing the dataset, we obtained 475 eligible location-networks and 132 eligible activity-networks and selected location-networks with probabilities greater than 0.1% as *LBMs* and activity-networks with probabilities greater than 0.5% as *ABMs*. Fig 3 depicts the *LBMs* and *ABMs* and their probabilities. The figure indicates that 99.35 and 98.46% of the total population can be characterized by 10 unique types of *LBMs* and *ABMs*, respectively. These high percentage values confirmed the heterogeneity in motif choices and the tendency to form distinctive motifs.

To quantify the properties of preferred motif choices, we plotted and fitted rank-ordered frequency distributions of motifs for three categories, i.e., *LBM*, *ABM*, and *JM*, via the least squares fit of the log-log transforms. We determined that all best-fitted distributions were **discrete generalized beta distributions (*DGBDs*)**, consisting of two polynomials (for more details, see **Methods**).

$$F(r)=Cr^{-\beta }(N+1-r{)}^{\gamma }$$ (1)

where *r* is the rank value, *N* is the maximum rank value, *C* is a normalization constant and *β* and *γ* are the two exponents. The Zipf’s law was expected when fitting the rank-frequency distributions, however, the *DGBD* outperformed Zipf’s law in describing scaling behaviors for the entire range because the *DGBD* has two exponents to control the curve of the distribution[42]. It was notable that the *DGBD* reduced to Zipf's law when *γ* = 0.

**Fig 4:**
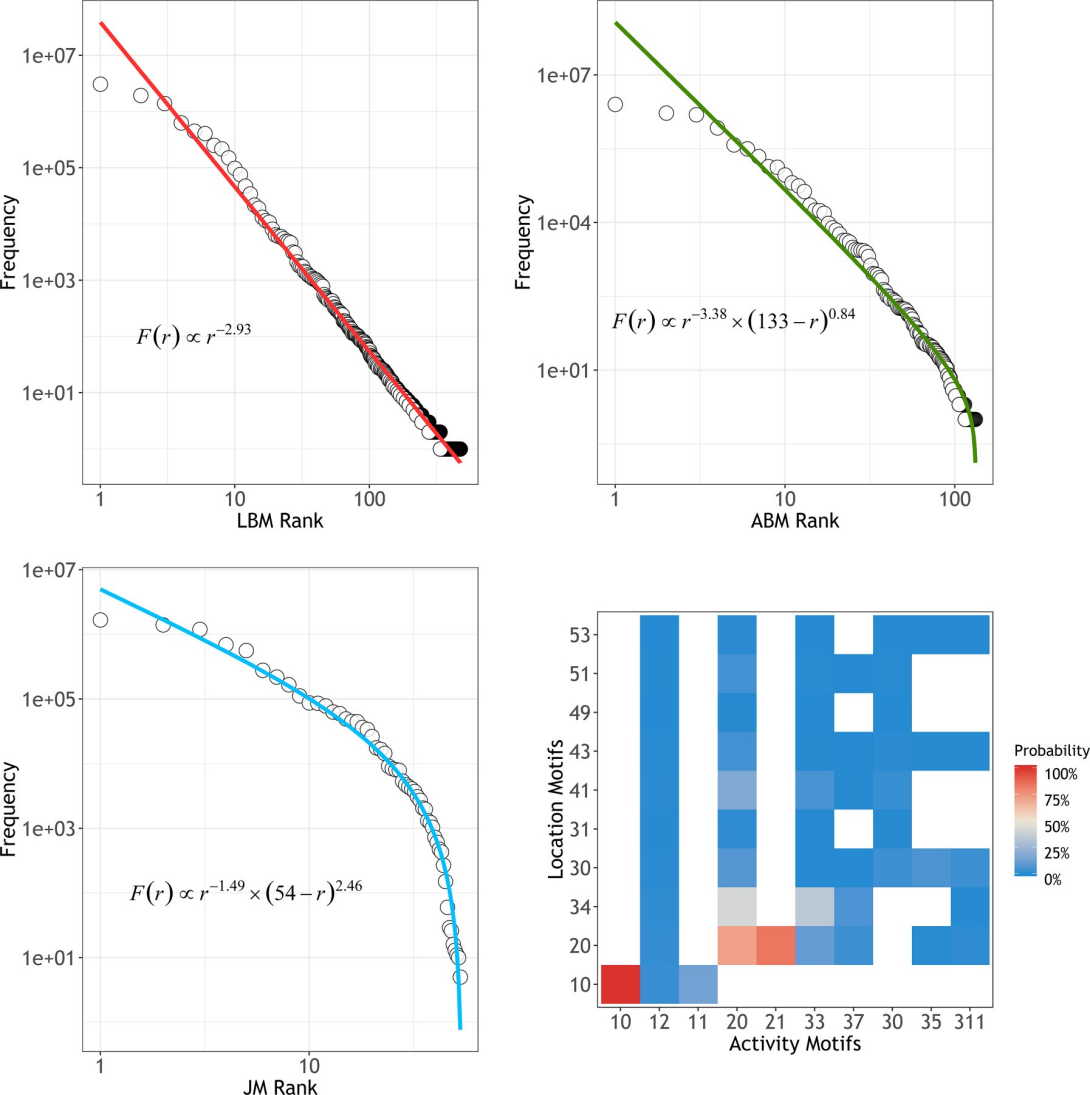
**Rank-frequency distributions for (a) location-based motifs, (b) activity-based motifs, and (c) joint motifs.** The hollow circles are the observed frequencies of rank values. The red, green and light blue lines denote the best-fitted distributions, *DGBDs*. The fitting was conducted via the least squares fit of log-transformed data. The corresponding function is also shown in each figure. **(d) The probability distribution of the joint motifs.** The white squares represent the absence of joint motifs. The red and pink squares indicate higher probabilities while the blue squares indicate lower probabilities.

We tested the statistical significance of *DGBD* fit using the *χ*^2^ test (chi-square test). We calculated *p-value* and found that, for each category, we cannot reject the null hypothesis that the empirical rank-ordered frequency distributions of motifs follow the *DGBD* at the significance level *p-val* = 0.05. As the **Table 1** shows, the *p-values* are 0.33, 0.43, and 0.46 for *LBMs*, *ABMs*, and *JMs*, larger than 0.05, meaning that the fit of the *DGBD* for *LBMs*, *ABMs*, and *JMs* all pass chi-square test, providing evidence for the quality-of-fit of the *DGBD*. **Fig 4** illustrates the best-fit of *DGBD F(r)* and the empirical rank-ordered motif frequencies.

**Table 1:** Fitted parameters (*β* and *γ*) of *DGBD*, sample size *N* (number of *LBMs*, *ABMs* and, *JMs*), and *p-values* for chi-square test.

Categories	*β*	*γ*	*N*	*p-value*
**LBMs**	2.93	-	475	0.3287
**ABMs**	3.38	0.84	132	0.4305
**JMs**	1.49	2.46	54	0.4564

**Fig 5:**
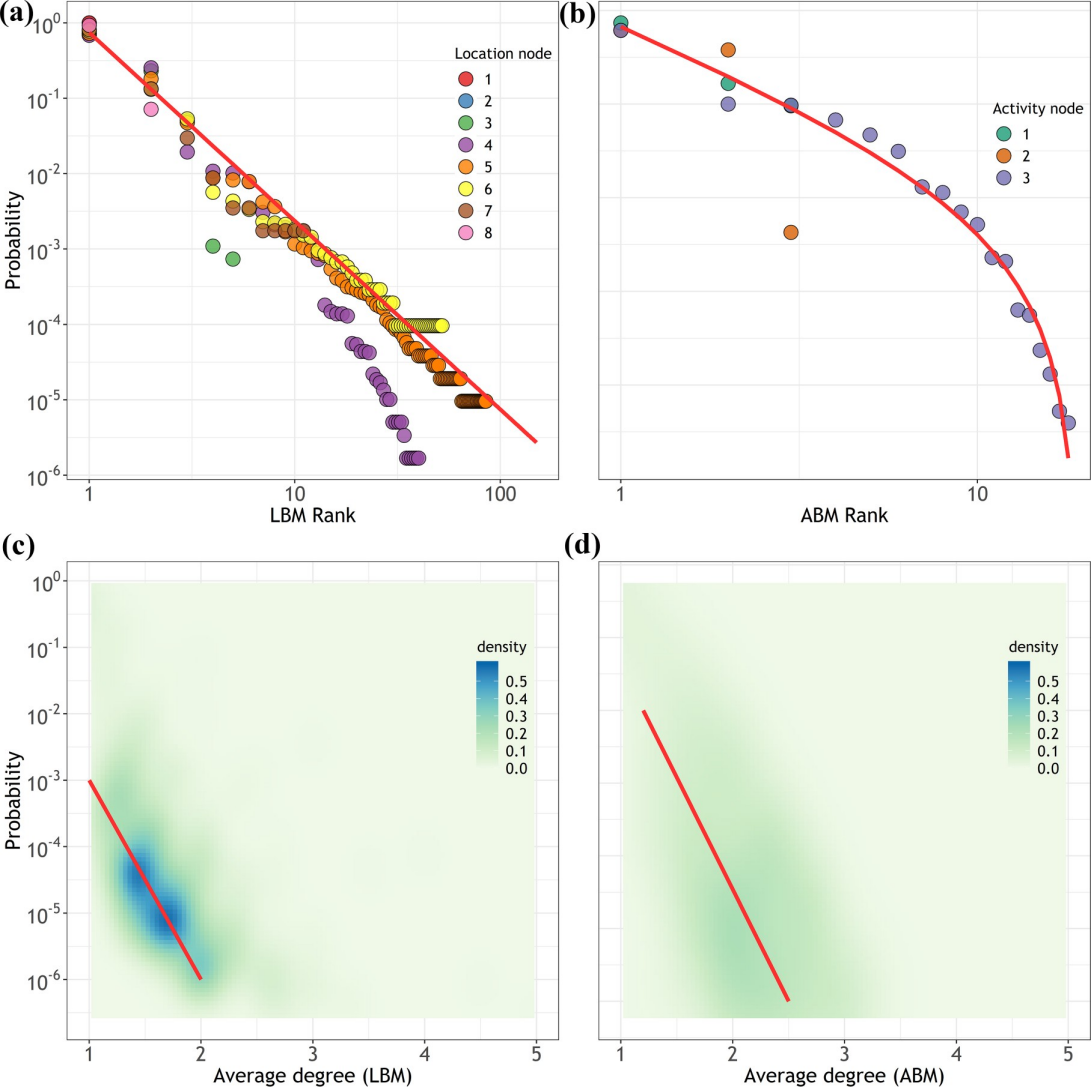
**(a)-(b) Rank-frequency distributions separated by different location nodes and activity nodes, respectively.** The different points in colors indicate the number of nodes, and the red lines represent the *DGBD* fit. **(c)-(d) Density maps of correlations of *F*(*r*)** and ⟨***k***⟩ **for the location-based and activity-based motifs, respectively**.

The fitted distributions of *LBMs*, *ABMs* and *JMs* obtained for *β* = 2.93, 3.38, and 1.49, respectively. The *β* determined the relative changes for small *r* values, which was related to the power-law behavior. The fitted distributions suggest that the daily travels had a high degree of regularity because of circadian rhythms. Certain motifs were always more popular and became fixed choices for those contributors; therefore, the fixed choices further skewed the distribution towards a power law. The different values of *β* indicated that the preferences for *ABMs* were more centralized, while those for *LBMs* and *JMs* were more spread; The *γ* controlled the tail skewness of the distribution. The larger the *γ*, the steeper the decay in the tail. The distribution of *LBMs* was fitted with *γ* = 0, and those of *ABMs* and *JMs* with *γ* = 0.84 and 2.46, respectively. This was because motif types are finite. It is natural to imagine that the motif types with large node were scarce because few people could travel to hundreds of locations in one day. We then fitted distributions separated by different nodes, and the results suggested that regardless of how many nodes (locations and activities) occurred in a day, the motif choices exhibited a strong similarity of rank-frequency distributions, as illustrated in Fig 5A and 5B.

Although the two exponents indicate independent significant meanings, we argued that there should be driving forces for certain increasingly popular motifs, which also made the frequency of inconspicuous motifs less significant than expected. We further hypothesized that cost efficiency is the substantial determinant in particular motif type to put into practice in a day. We use the average degree ⟨*k*⟩ of a motif as a proxy of cost efficiency, which is defined by:

$$\langle k\rangle =\frac{E}{N}$$ (2)

Where *E* is the number of edges and *N* is the number of nodes. For instance, if one person plans to visit three distinct locations, the most effective way is a round trip; thus, only three trips need to be conducted, in which case, ⟨*k*⟩ is equal to 1. If he or she moves multiple times between nodes, the value of ⟨*k*⟩ is larger than 1. The higher the ⟨*k*⟩, the less efficiently the individual travels.

We examined the correlation between the frequencies of motifs, *F*(*r*), and their cost efficiencies, ⟨*k*⟩, for the *LBMs* and *ABMs*. Fig 5C and 5D show the corresponding density maps. The negative correlations between *F*(*r*) and ⟨*k*⟩ indicate that the individuals prefer the motif with high efficiency rather than low efficiency. The results concluded that there might be a principle behind it to result in such choices. Combined with the fitted *DGBDs*, we proved that the frequency distributions of motifs were the "need" distributions determined by how often choosing as motifs and why some were more popular, and that the hidden least effort principle[48] drives human travels, which means although individuals plan their travels with unique propensities, such as specific travel purposes, they always tend to choose the most convenient way, at the same time, that satisfy their needs. The densities of the *LBMs* were more concentrated, thus leading to a significantly higher value of *β* compared to the *ABMs*. The finding was also consistent with a variety of contexts characterized by Zipf’s law or power-law, such as the preferential attachment in networks[49] and city sizes[50], the 80–20 rule in income distributions[51].

### Scaling properties of the average travel distance

We investigated the average travel distance in a motif. The average travel distance *D*_*ave*_ is a comprehensive measure that reflects factors considerable when people choose their motif types. *D*_*ave*_ was calculated as the sum of the Euclidian distance between each pair of consecutive nodes divided by the number of edges in a motif.

$$D_{\mathrm{ave}}=\frac{\sum_{j=1}^{N-1}d_{j,j+1}}{E}$$ (3)

**Fig 6:**
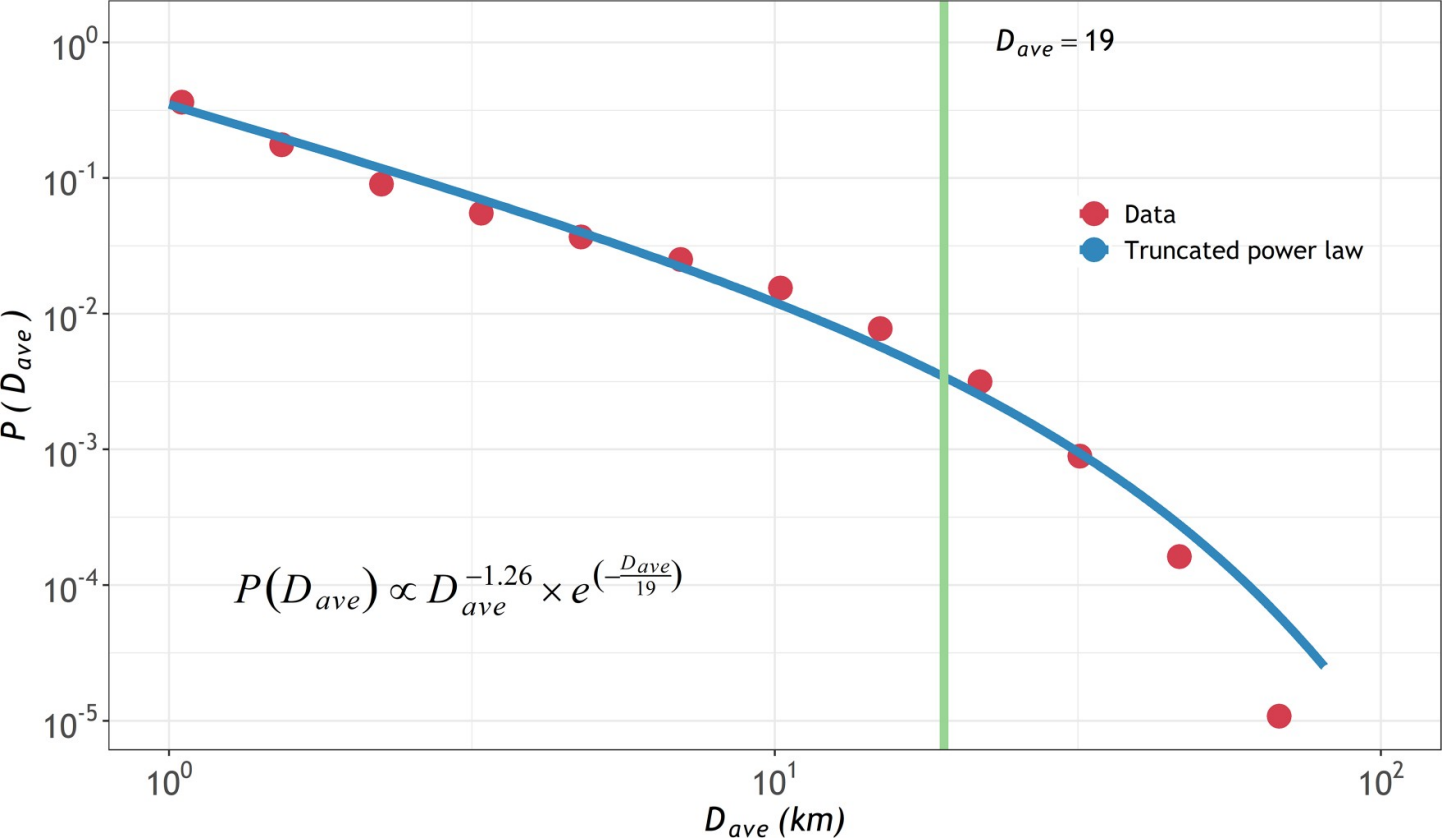
The probability distribution of *D*_*ave*_ for the overall travels in the data. The solid blue line represents the power law with an exponential cut-off fit, of which the functional form is shown as well, while the red points refer to the log-transformed data. The vertical green line indicates the cut-off value *κ*. It should be noted that the log-transformation is only for visualize data but not for fit data.

Here *j* represents the consecutive node in a motif. We first quantified the scaling properties by fitting the ensemble probability distribution of *D*_*ave*_ for the entire population. The statistical fitting was carried out by the maximum likelihood estimation and the statistical significance test was performed by a bootstrap-Kolmogorov-Smirnov approach (see Methods for details). As **Fig 6** shows, we found that the power law with an exponential cutoff (or called the exponential truncated power law) was the best PDF. The *p-value* of goodness of fit was 0.87, larger than 0.10. In contrast, three other distributions, including the log- normal, exponential, and pure power law, were also fitted and are illustrated in **S1 Text**.

The exponential truncated power law in which a power law is multiplied by an exponent is given by

$$P(D_{ave})={{C*D}_{ave}}^{-\alpha }*e^{\frac{-D_{ave}}{\kappa }}$$ (4)

Here, *C* is the normalization constant, *α* is the scaling parameter and *κ* is the cut-off parameter. The parameters simultaneously control the shape of the distribution, which starts out as a power law and ends up as an exponential distribution. The fitted *α* value was 1.26, which was in agreement with existing studies, i.e., *α* = 1.55 [6] and 1.25[7] using CDRs, and 1.57[15] and 1.39[17] using GPS trajectories, although these data covered different populations at various scales. The fitted cut-off parameter *κ* was 19 km.

We then analyzed the physical significance of the scaling and cut-off parameters to determine the potential impacts on motif behavior. Within a short range of *D*_*ave*_ before the cut-off *κ*, people would not treat their potential *D*_*ave*_ as a restrictive factor, but they had strong preferences for visiting places for specific activities despite the distance. This phenomenon was reflected by the power-law behavior of abundant resources required for engaging in activities. The decaying degree of the power law is represented by *α*. A smaller *α* means a slower decrease with a wider spatial diffusive range, while a larger *α* indicates a faster decrease with a narrower spatial diffusive range. Once the distance exceeded a threshold, i.e., the cut-off value *κ*, people may hesitate to engage in long-distance traveling. The distribution, therefore, decayed faster (exponentially) than the power law, which increased the possibility of the distribution turning into a normal diffusive process. A smaller *κ* indicated a shorter power-law range and a longer exponential tail. *κ* denoted the breakpoint between the two processes. In other words, different *κ* values represented the abilities to break through resource limitations. Therefore, we concluded that the distribution of *D*_*ave*_ with exponential truncated power-law behaviors was caused by the combination of adequate activity resources (significance of *α*) and varied diffusion limitations characterized by mobility scenarios, such as the travel costs, geographic boundaries, and mobility regularities (significance of *κ*).

### Distance impacts on the motifs

Because distance scaling contributes to explaining the mechanisms of motif travels, a natural question was proposed regarding how distance affected the popularities of motif types. To examine this point, we grouped the overall population according to the node numbers and motif types and fitted the distance distributions for each group separately to test whether they had the same scaling properties.

**Fig 7:**
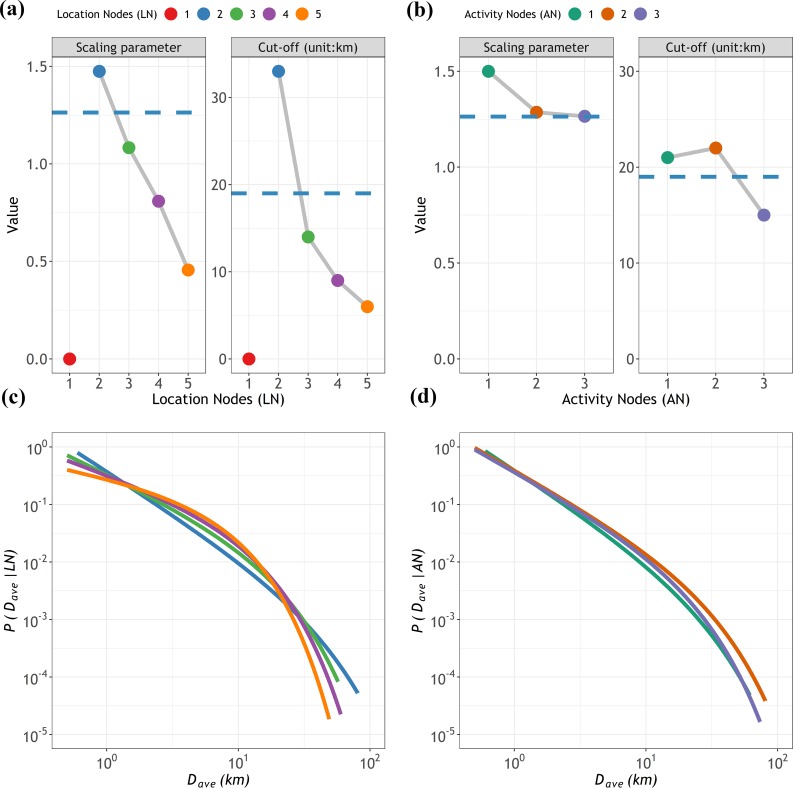
The probability distributions of *D*_*ave*_ and parameters at the node level. (a) and (c) for the *LN* set and (b) and (d) for the *AN* set. The different colors represent corresponding *LN* values or *AN* values, as shown on the top legend. As the group with *LN* = 1 represents those individuals who only have visited one location in one day, their parameters are always expressed as zero. The dashed horizontal line in (a) and (b) indicates the parameter values for the ensemble distribution, as referred to in Fig 5. The solid lines in (c) and (d) represent the power law with an exponential cut-off fit for each group of the *LN* set and *AN* set.

**Fig 7** shows the best scaling curves and corresponding parameters of *p*(*D*_*ave*_|*LN*) and *p*(*D*_*ave*_|*AN*). We found that all the fits passed the bootstrap-K-S test for the goodness of fit. A detailed summary of the fitted results is shown in S1 Text. It was observed that, even if all of these groups were best described by exponential truncated power laws, they still exhibited different significances. In summary, the linear decrease in *α* with *LN* in *p*(*D*_*ave*_|*LN*) (Fig 7A) suggested that visiting more locations in a day required a wider spatial diffusive range to find for more resources, while the decreasing trend in *p*(*D*_*ave*_|*AN*) (Fig 7B) were not so obvious compared to the *LN* set. The one-*AN* and two-*ANs* groups got almost the same *α* values, implying that the locations are more influential than activities on distance scalings. If a group had a large *κ*, it implied that people in this group generally have higher tolerability of traveling long distances to meet their needs. *κ* decreased as *LN* increased, while this trend could not be observed in the *AN* set. The two-*AN* group had the highest value of *κ*. The reason is that most people with two-*ANs* had one home activity, which usually occurred at a fixed location; thus, people in this group had a relatively fixed travel distance. In contrast, the one-*AN* group included social activities that were engaged in at multiple alternative locations without in-home or working activities; thus, people in this group chose unfixed locations with lower tolerances in longer traveled distance. The scaling relations with *LN* and *AN* sets indicated that the more locations were to be visited, the more activities were to be engaged in, leading to more resources needed and therefore the limitations were reached sooner. More importantly, the popularities of groups, namely, the frequencies of motifs, were all positively correlated with their scaling values. This result sustains the finding that scaling differences were attributed to motif choices.

**Fig 8:**
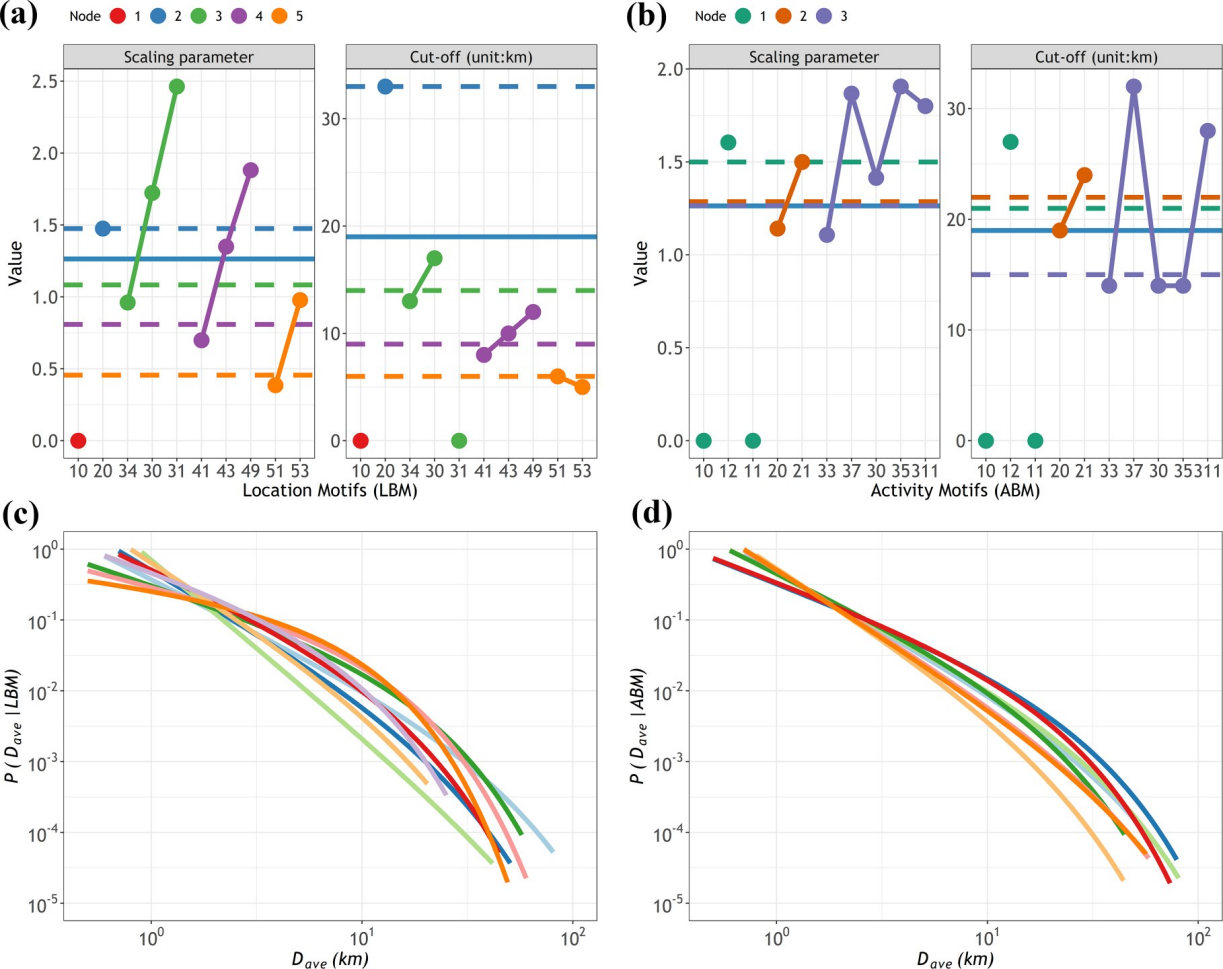
The probability distributions of *D*_*ave*_ and parameters at the motif level. (a) and (c) for the *LBM* set and (b) and (d) for the *ABM* set. The different colors represent corresponding *LN* values or *AN* values, as shown on the top legend. Because certain groups have no *D*_*ave*_ data, their parameters are always expressed as zero. The dashed horizontal lines in (a) and (b) indicate the parameter values for the *LN* set and *AN* set, respectively, as shown in Fig 7, and the blue solid line represents the parameter values for the ensemble distribution. The solid lines in (c) and (d) represent the curves of the best-fitted distributions for each group of *LBM* and *ABM*, some of which are the power law with an exponential cut-off, and some are the pure power law. For instance, *LBM* 31 is fitted with a pure power law with *α* = 2.46.

**Fig 8** presents the results for *p*(*D*_*ave*_|*LBM*) and *p*(*D*_*ave*_|*ABM*). We also found that all the fits passed the bootstrap-K-S test for the goodness of fit. A detailed summary of the fitted results is shown in S1 Text. Unlike the fitting at the node level, the result indicated that certain best-fitted distributions were achieved by the power law rather than the truncated power law, such as *LBM* 31. Similarly, *α* for different *LBMs* belonging to the same *LN* set showed a decreasing trend with corresponding frequencies (**Fig 8A**), while this trend could not be clearly observed in the *ABM* set, especially in the three-*AN* group (**Fig 8B**). Indeed, the motifs in the *ABM* set shared similar percentages of the population, and their activity orders exhibited no essential discrepancies. The differences in *κ* also suggested that the lower the *κ*, the more popular the motifs.

As discussed above, the statistical properties describing *p*(*D*_*ave*_) conditional on the different groups confirmed that the *D*_*ave*_ distributions of different motifs obeyed similar scaling laws. The visited locations, activity purposes, and motif types affected the scaling parameters of the distribution but not its scaling form. Therefore, our results suggested that the scaling laws in distance were regulated by certain mechanisms that are statistically universal. The scaling parameters coincided with the popularities of motifs, suggesting that distance impacted motif choices. It is not difficult to imagine that motif choices were induced by travel self-adaptive systems in which people were unwilling to diffuse with wider spatial ranges unless they were compelled to do so when optimizing their daily travels for working, shopping, sports, entertainment, etc.

## Discussion

The abstraction of human travel into network-based structures advances the clear understanding of highly heterogeneous human behaviors, as uniform measurements are absent when modeling human travel. The limited quantities of location-based and activity-based motifs suggested that, although human travel seems chaotic, it is highly predictable and can be well represented in a structural way. We focused on the quantification of motif choices based on statistical properties. In particular, both location-based and activity-based motif distributions were characterized by rank-frequency distributions following the *DGBD* model. The empirical distributions, as well as their fitted parameters, provided a deeper understanding of motif choices. The results suggested that the least effort principle is the fundamental law that gives rise to the *DGBD* model. The least effort principle surfaces in a multitude of natural and social systems, especially as a driving force of human behaviors. Our results verified that this principle existed in the daily travels of people.

Our approach further investigated the scaling properties of the average travel distance behind motifs. The scaling form, namely, the exponential truncated power law, suggested that both adequate activity resources and cost limitations drove travels. In addition, the scaling forms were invariant for all node numbers and motif types, and the values of parameters coincided with the popularities of motifs, suggesting that the distance distributions for all motif types could be characterized by statistically universal mechanisms. The scaling differences revealed that potential travel self-adaptive patterns were inherent. The linkage of scaling parameters in distance distributions and their physical significance has successfully expressed human mobility as quantitative physics models and explained the travel choices with behavioral dynamics.

These results not only deepen the insights into human life in cities but also demonstrate the use of new mobility data as proxies for human travel. It is expected that these results will be used to forecast high-precision human behavioral changes, with several applications in traffic management and emergency response. There are still some limitations to the current study. First, one-day mobile phone positioning data were used. Although human activities hold the regularity, long-time data should be collected and further verified our findings. Second, the interaction between motifs and geographical context contribute to the motif choices. Previous studies demonstrate the mechanism of the interaction is complex. The question of urban factors affecting motif choices should be investigated in the future.

## Supporting information

S1 Text Supporting figures and tables. (PDF)

S1 Data Public data. (ZIP)
