# Supplementary material for: Characterizing preferred motif choices and distance impacts
Source: PLoS One. 2019 Apr 16;14(4):e0215242. doi: 10.1371/journal.pone.0215242 (PMC6467417; doi:10.1371/journal.pone.0215242)
Supplement: S1 Text — (PDF) [file pone.0215242.s001.pdf]

Supporting information S1 Text

Table of contents:

Figure S1. The daily trajectory from mobile phone location data. Page S3

Figure S2. Spatio-temporal patterns of stays and activities detected from data. Page S4

Figure S3. Individual motif travel patterns Page S5

Figure S4. D_{ave} distribution of overall travels and four fitted distributions Page S6

Figure S5. D_{ave} distributions for aggregations of *LNs* Page S7

Figure S6. D_{ave} distributions for aggregations of *ANs* Page S8

Figure S7. D_{ave} distributions for aggregations of *LBMs* Page S9

Figure S8. D_{ave} distributions for aggregations of *ABMs* Page S10

Figure S9. Bootstrap-K–S test for overall data. Page S11

Figure S10. Bootstrap-K–S test for aggregations of *LNs* Page S12

Figure S11 Bootstrap-K–S test for aggregations of *ANs* Page S13

Figure S12. Bootstrap-K–S test for aggregations of *LBMs* Page S14

Figure S13 Bootstrap-K–S test for aggregations of *ABMs* Page S15

Table S1. Fitted distributions Page S16

Table S2. The detailed summary of the fitted results for D_{ave} distributions at the node-level Page S17

Table S3. The detailed summary of the fitted results for D_{ave} distributions at the motif-level Page S18

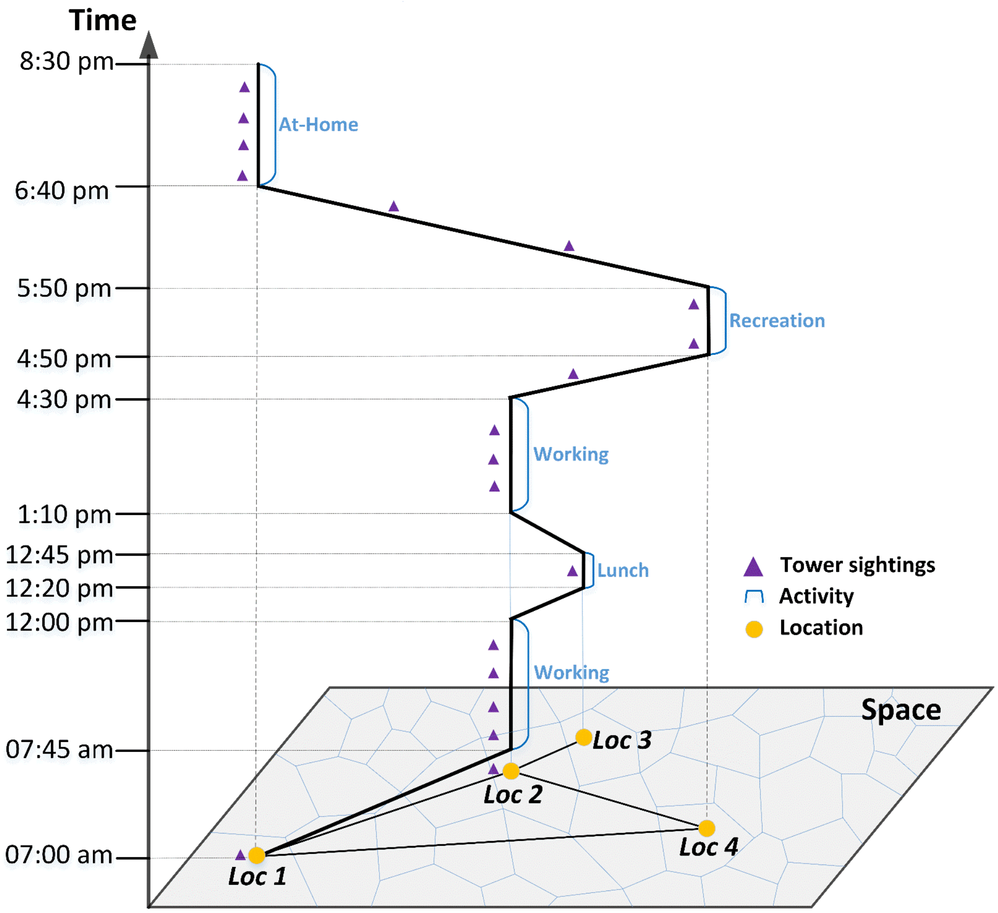

Figure S1. The daily trajectory from mobile phone location data.

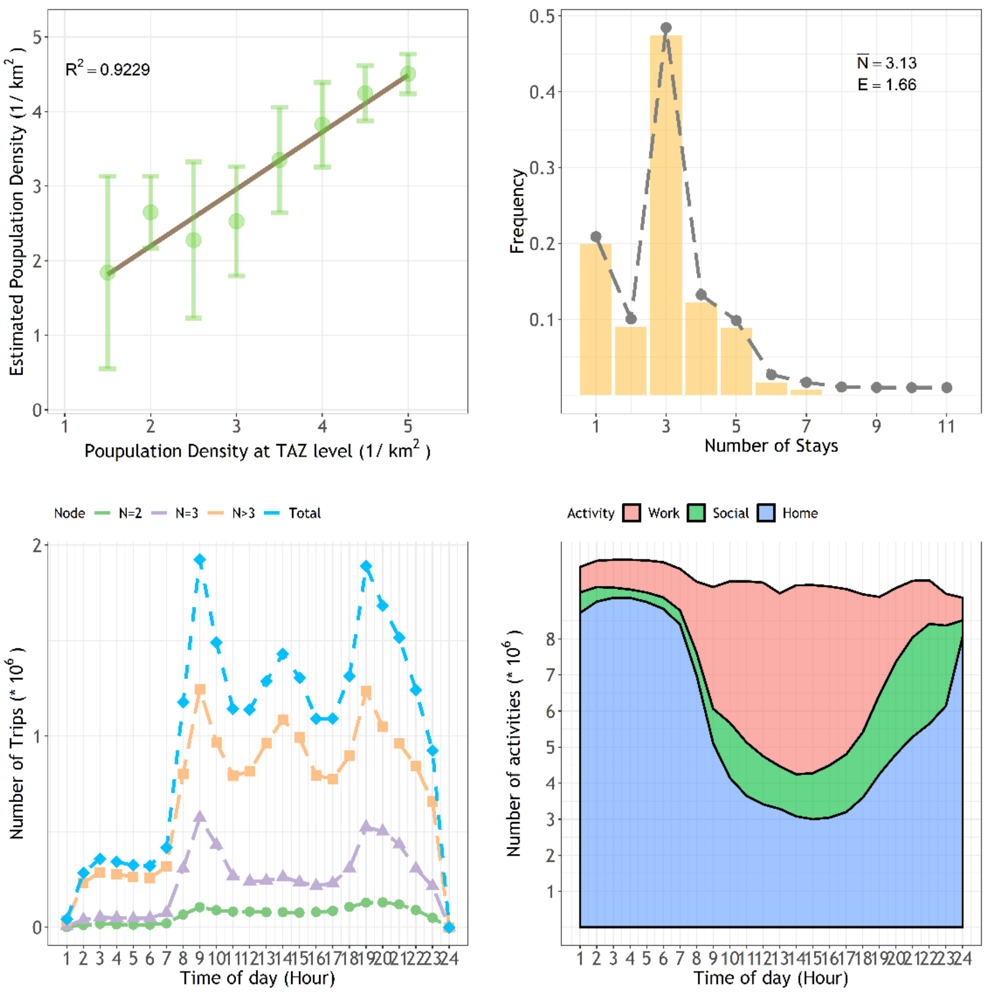

Figure S2. Spatio-temporal patterns of stays and activities detected from data.

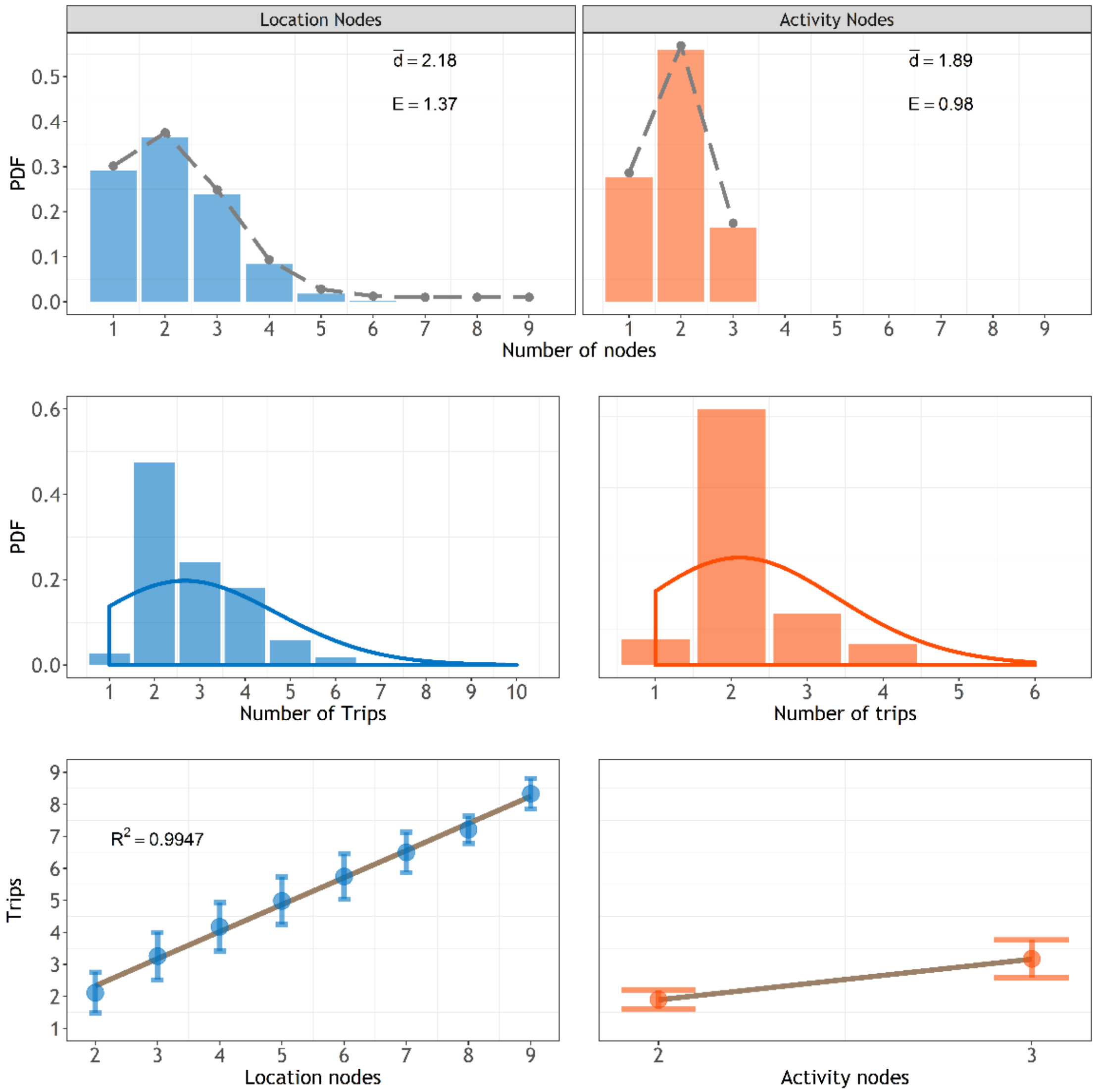

Figure S3. Individual motif travel patterns

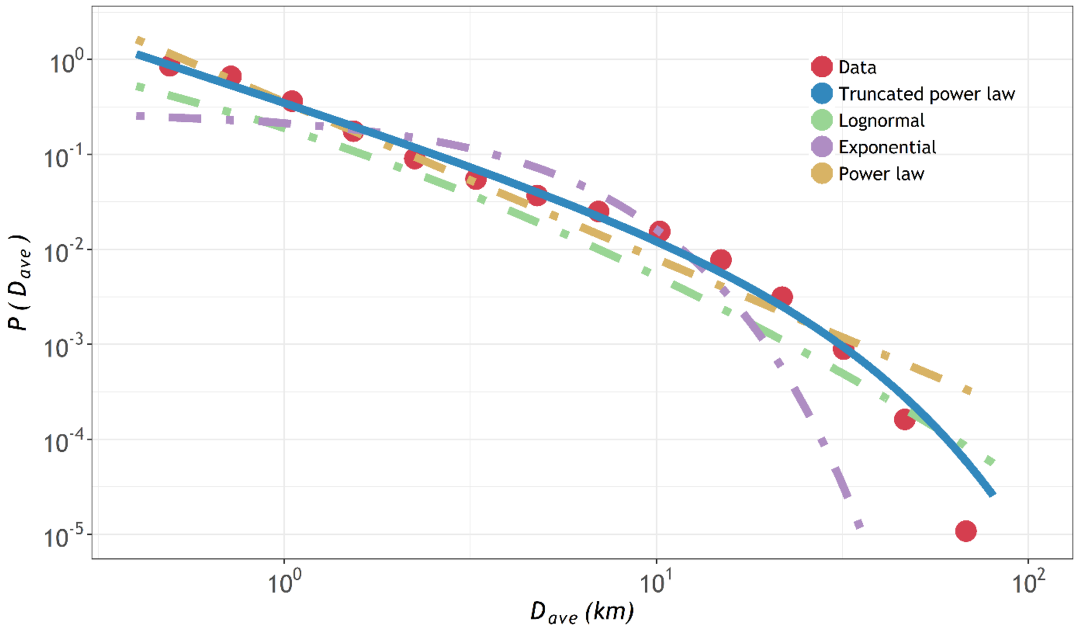

Figure S4. D_{ave} distribution of overall travels and four fitted distributions. The solid blue line represents the best fitted distribution, i.e., power law with the exponential cutoff fit, while the red points refer to log-transformed data. The green, purple, and yellow dashed lines indicate lognormal, exponential and power law distributions, respectively.

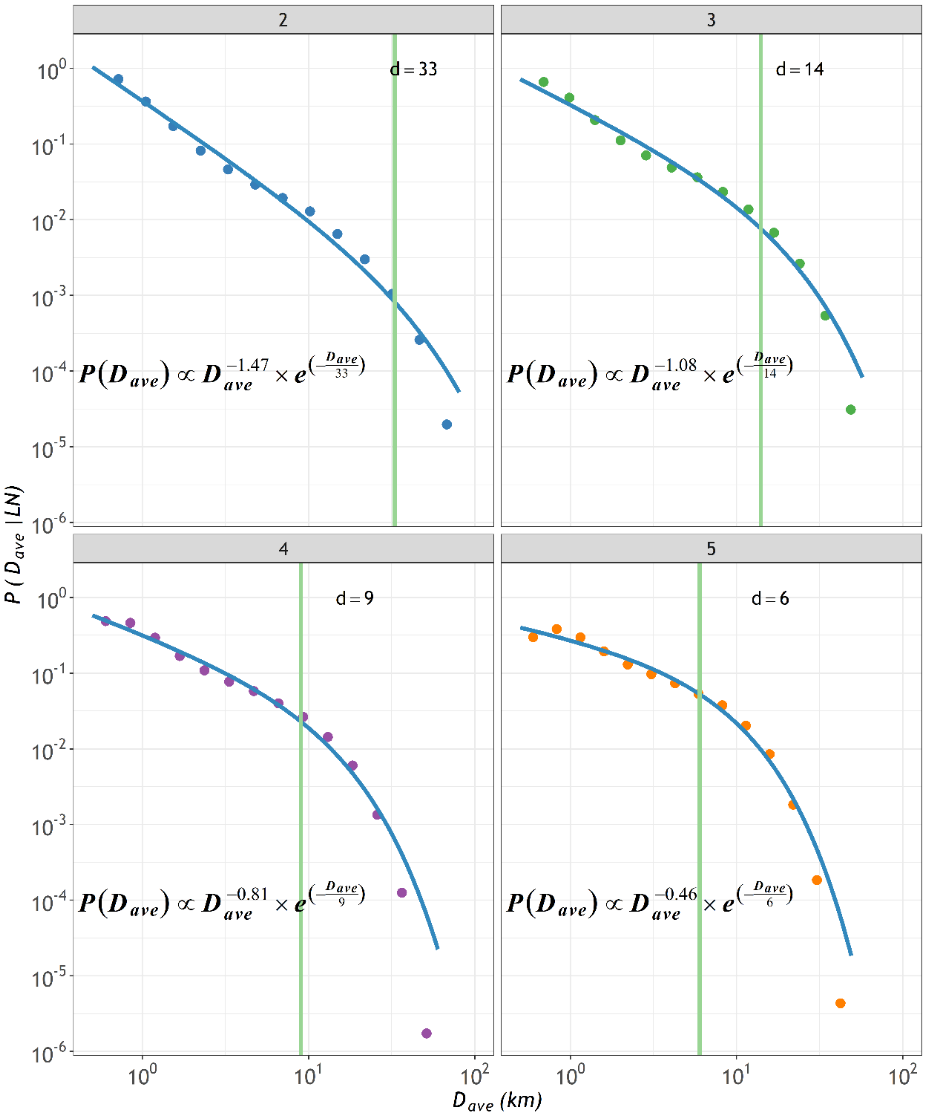

Figure S5. D_{ave} distributions for aggregations of *LN* ((a) node 2, (b) node 3, (c) node 4 and (d) node 5). The solid blue line represents the best-fitted distribution, i.e., the power law with an exponential cutoff, where the dotted points refer to log-transformed data. The vertical green lines indicate corresponding values of the cut-off value κ in fitted distributions.

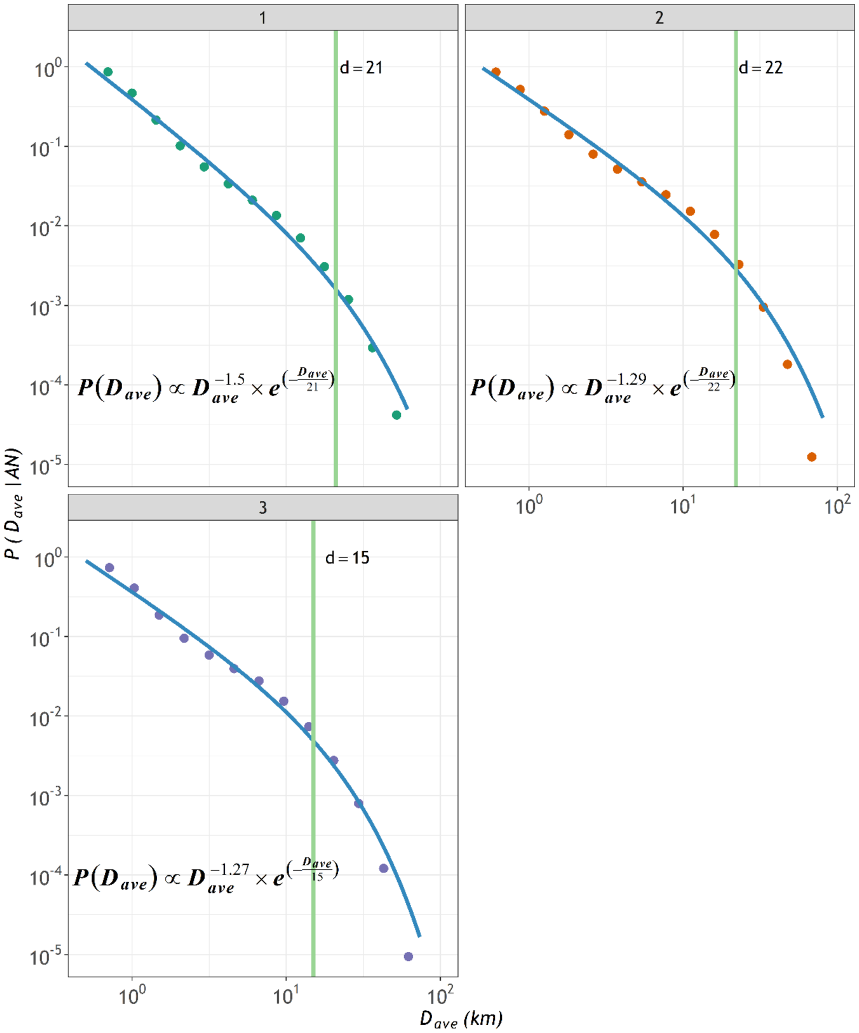

Figure S6. D_{ave} distributions for aggregations of AN ((a) node 1, (b) node 2, and (c) node 3). The solid blue line represents the best-fitted distribution, i.e., the power law with an exponential cutoff, where the dotted points refer to log-transformed data. The vertical green lines indicate corresponding values of the cut-off value κ in fitted distributions.

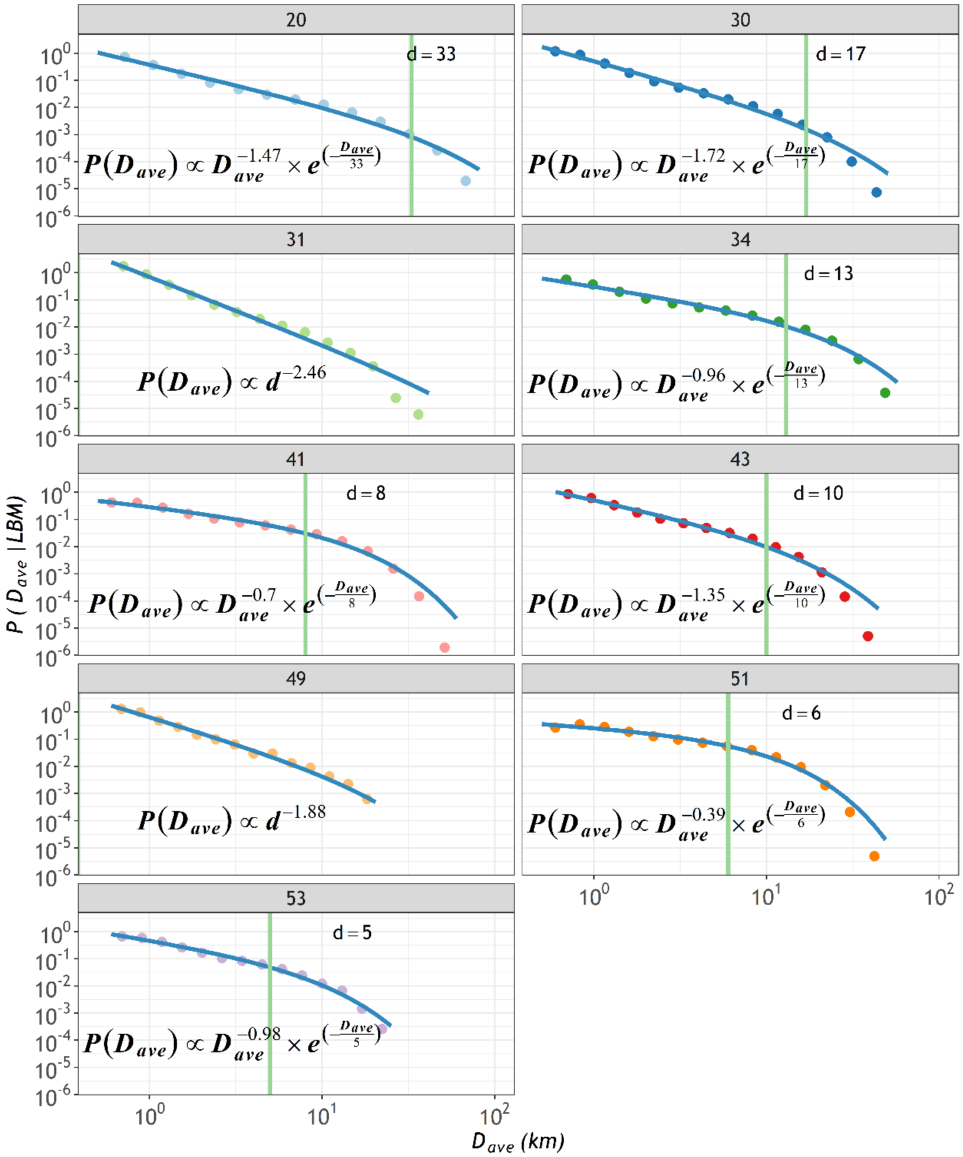

Figure S7. D_{ave} distributions for aggregations of *LBM*. Their motif ids are displayed at the top of each subplot. The solid blue line represents the best-fitted distribution, i.e., pure power law or exponential truncated power law, where the dotted points refer to log-transformed data. The vertical green lines indicate corresponding values of the cut-off value κ in fitted distributions of the exponential truncated power law. The *LBM* 31 and 49 shows pure power law with α=2.46 and 1.88.

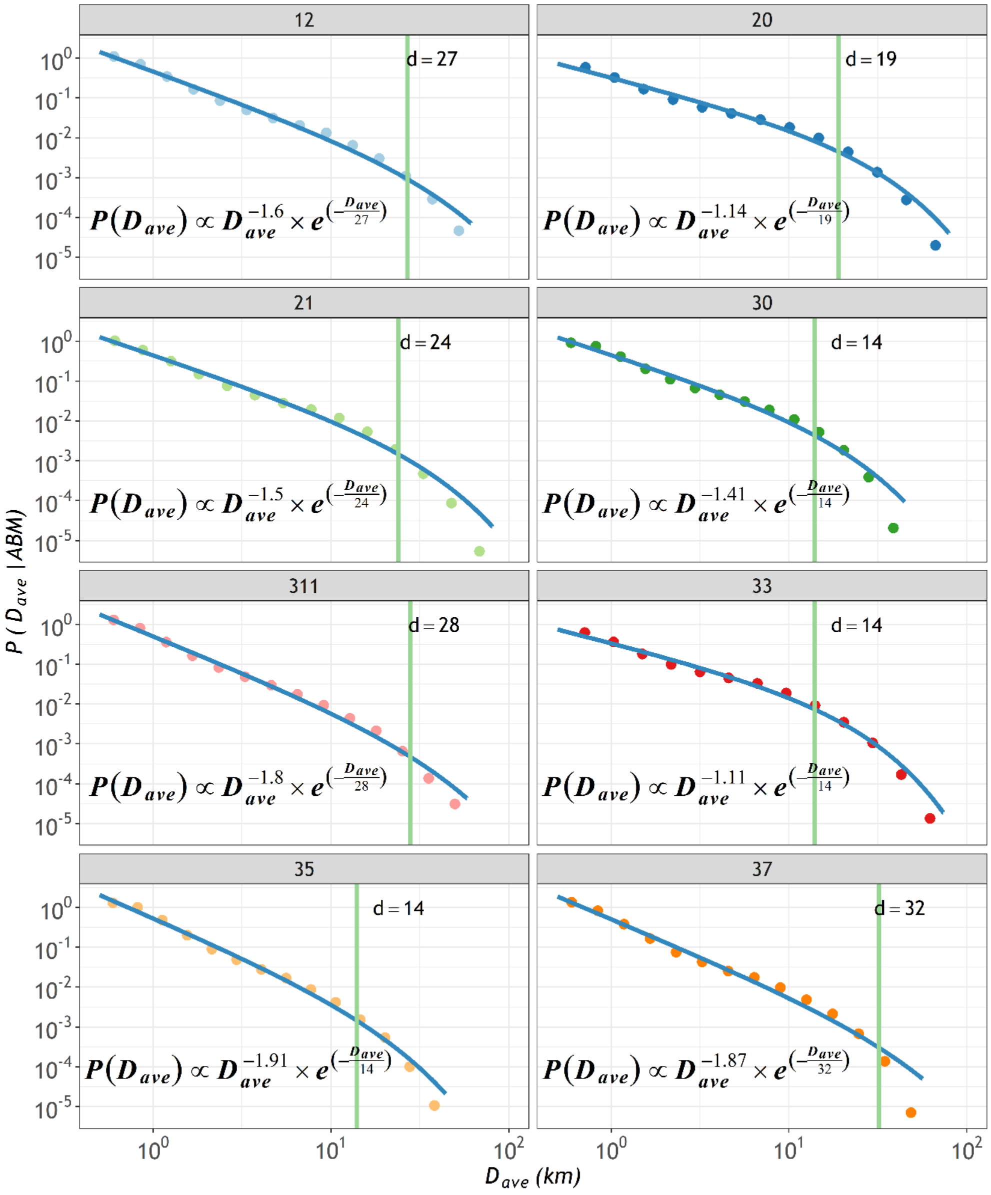

Figure S8. D_{ave} distributions for aggregations of ABM. Their motif ids are displayed at the top of each subplot. The solid blue line represents the best fitted distribution, i.e., pure power law or exponential truncated power law, where the dotted points refer to log-transformed data. The vertical green lines indicate corresponding values of the cut-off value κ in fitted distributions of the exponential truncated power law.

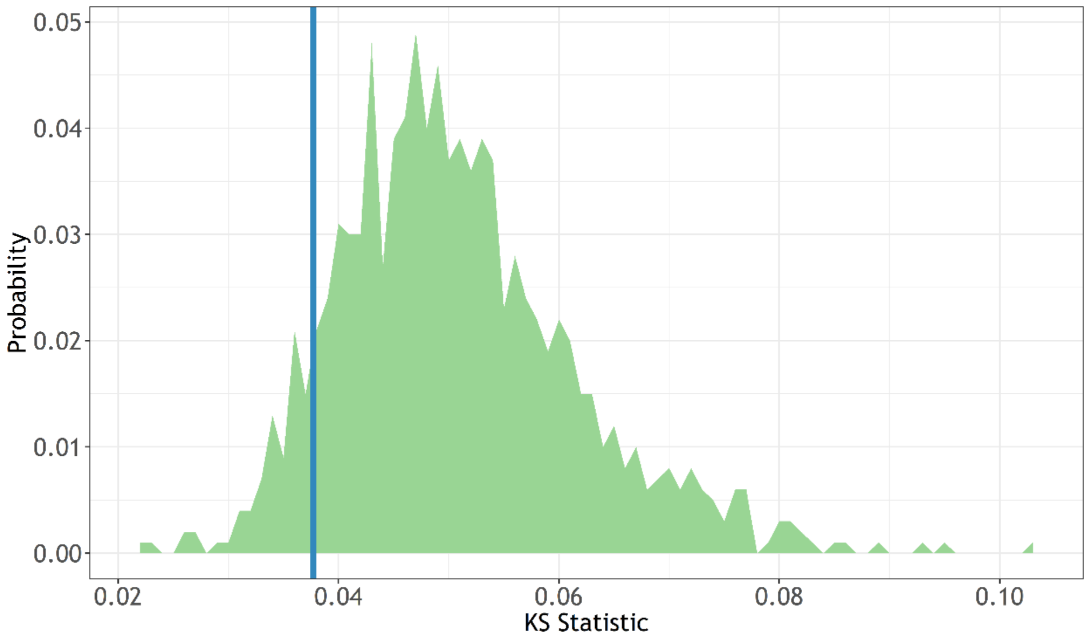

Figure S9. Bootstrap-K–S test for overall data. The vertical blue line represents the KS statistic D^* obtained for the empirical data. The green area indicates $P(D)$ obtained for synthetic data generated with 2500 bootstrapping.

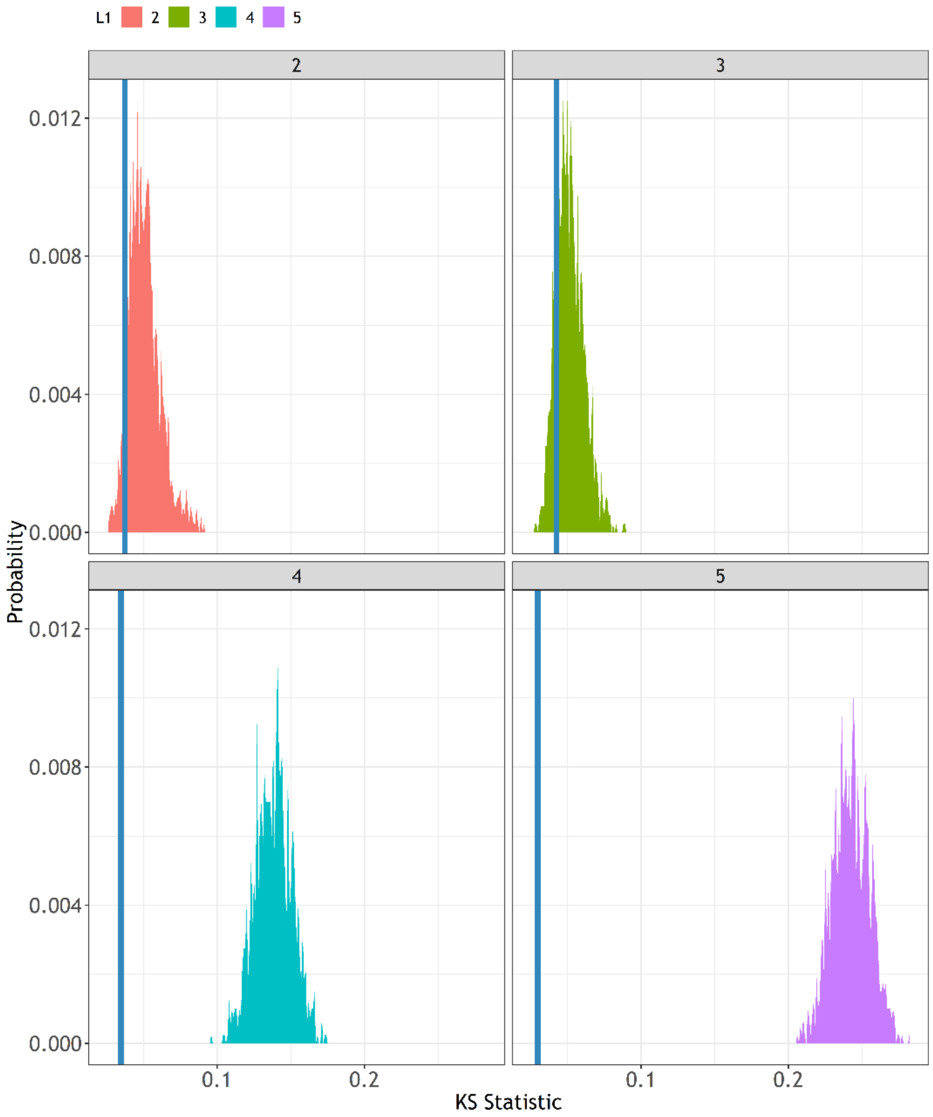

Figure S10. Bootstrap-K–S test for aggregations of *LN* ((a) node 2, (b) node 3, (c) node 4 and (d) node 5). The vertical blue line represents the KS statistic *D** obtained for the empirical data. The area indicates *P(D)* obtained for synthetic data generated with 2500 bootstrapping.

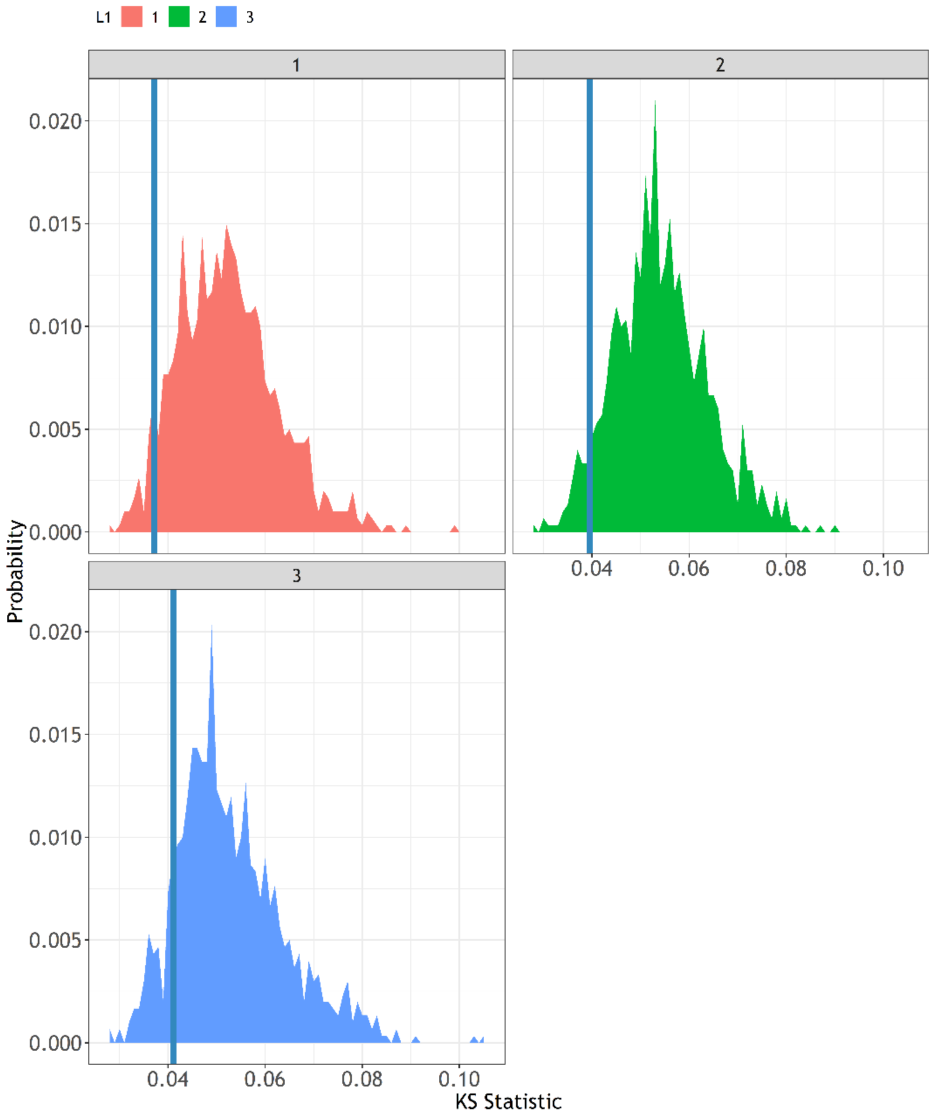

Figure S11. Bootstrap-K–S test for aggregations of *AN* ((a) node 1, (b) node 2, and (c) node 3). The vertical blue line represents the KS statistic *D** obtained for the empirical data. The area indicates *P(D)* obtained for synthetic data generated with 2500 bootstrapping.

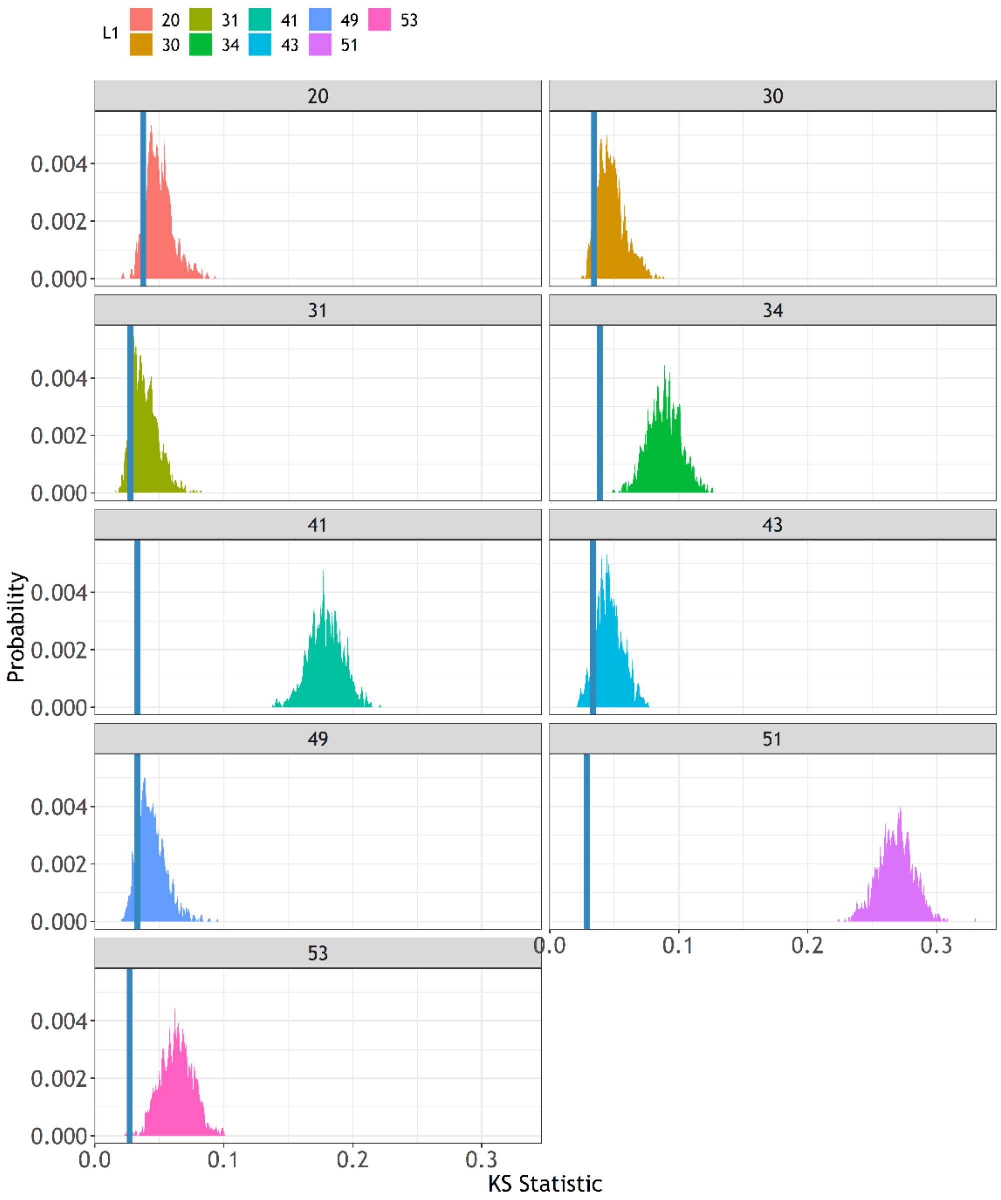

Figure S12. Bootstrap-K–S test for aggregations of *LBM*. Their motif ids are displayed at the top of each subplot. The vertical blue line represents the KS statistic *D** obtained for the empirical data. The area indicates *P(D)* obtained for synthetic data generated with 2500 bootstrapping.

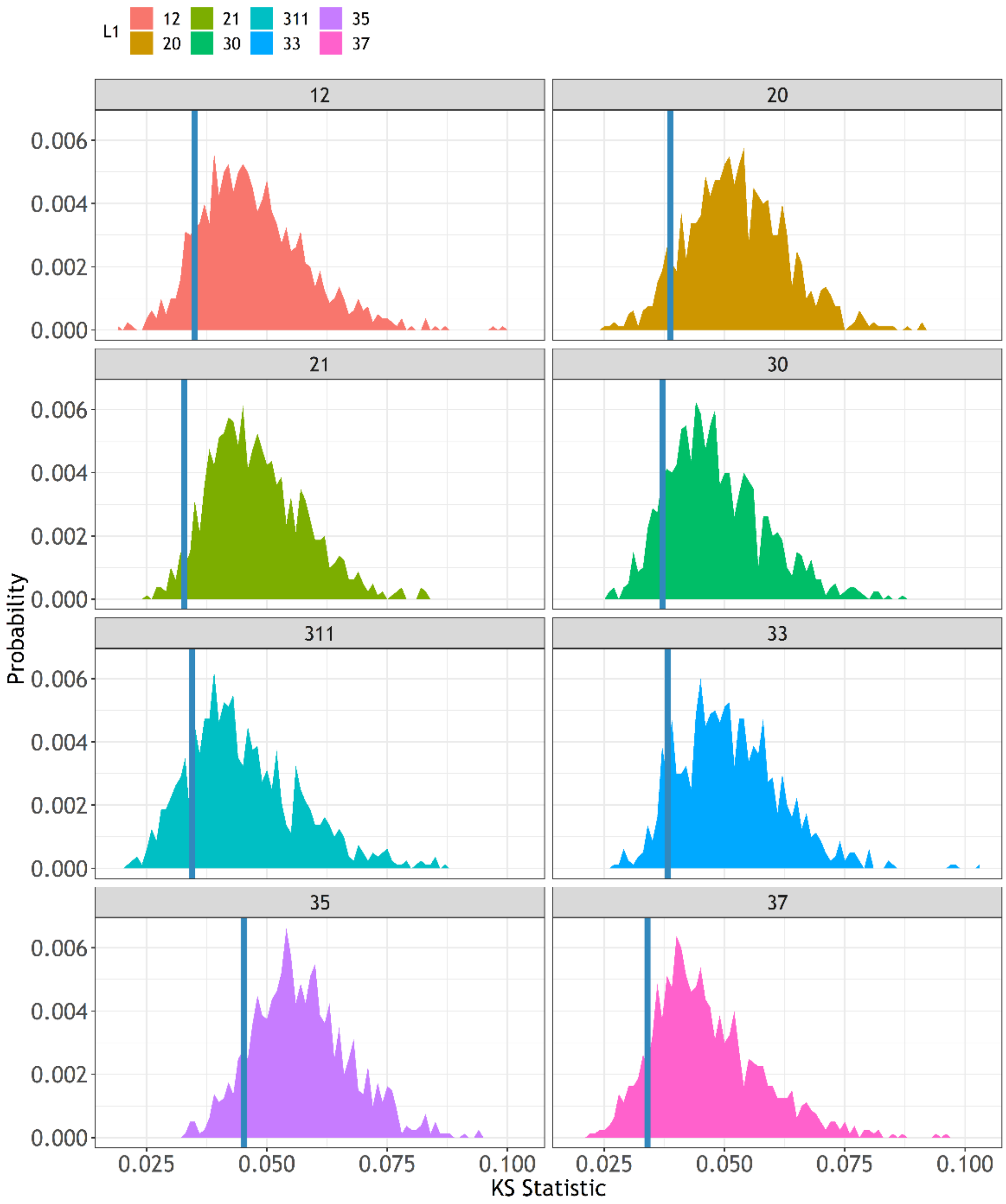

Figure S13. Bootstrap-K–S test for aggregations of *ABM*. Their motif ids are displayed at the top of each subplot. The vertical blue line represents the KS statistic *D** obtained for the empirical data. The area indicates *P(D)* obtained for synthetic data generated with 2500 bootstrapping.

Table S1. Fitted distributions.

Fitted distribution	Probability distribution function(PDF)
Exponential truncated power law	$Cx^{-\alpha}e^{-\frac{x}{\kappa}}$
Lognormal	$\frac{1}{x\sigma\sqrt{2\pi}}exp\left[-\frac{(ln(x)-\mu)^2}{2\sigma^2}\right]$
Power law	$Cx^{-\alpha}$
Exponential	$\lambda e^{-\lambda x}$

Table S2. The detailed summary of fitting results of D_{ave} distributions at the node-level. For each group, we show the number of node, the number of users observed for this group (N_{total}), the number of users observed larger than κ (N_{tail}), the proportion of users in the tail region, lower bound to the power-law-like fit ($D_{\mathrm{fit\_min}}$), maximum value of empirical data (D_{emp_max}), the K-S statistic(D), the *p-value* of K-S bootstrapping test, estimated exponent (α), estimated cut-off value (κ), first moment (mean), second moment (standard deviation), third moment (skewness), forth moment (kurtosis), and best fitted distribution. TP represents exponential truncated power law, and P represents power law.

Type	*Node*	N_{total}	N_{tail}	N_{tail}/N_{total}	$D_{\mathrm{fit\_min}}$	D_{emp_max}	D	*p-value*	α	κ	Mean	Standard deviation	Skewness	Kurtosis	Fitted distribution
Overall	-	4831160	162438	3.4%	0.4	80.64	0.038	0.91	1.26	19	3.89	5.87	3.14	13.36	TP
Location Nodes	2	2636765	22702	0.9%	0.4	80.64	0.037	0.927	1.47	33	3.63	6.20	3.56	16.36	TP
	3	1633717	110530	6.8%	0.4	57.27	0.042	0.844	1.08	14	4.12	5.57	2.53	7.73	TP
	4	463459	73738	15.9%	0.5	60.04	0.035	1	0.81	9	4.56	5.07	1.99	4.67	TP
	5	83964	23213	27.6%	0.5	49.05	0.030	1	0.46	6	4.57	4.47	1.73	3.57	TP
Activity Nodes	1	67197	993	1.5%	0.4	61.27	0.037	0.949	1.50	21	2.83	4.70	3.91	20.70	TP
	2	3281034	92161	2.8%	0.5	80.64	0.039	0.947	1.29	22	4.40	6.30	2.93	11.55	TP
	3	1173572	46644	4.0%	0.4	73.52	0.041	0.888	1.27	15	3.41	5.01	3.24	14.76	TP

Table S3. The detailed summary of fitting results of D_{ave} distributions at the motif-level. For each group, we show the number of node, the number of users observed for this node (N_{total}), the number of users observed larger than κ (N_{tail}), the proportion of users in the tail region, lower bound to the power-law-like fit ($D_{\mathrm{fit\_min}}$), maximum value of empirical data (D_{emp_max}), the K-S statistic(D), the *p-value* of K-S bootstrapping test, estimated exponent (α), estimated cut-off value (κ), first moment (mean), second moment (standard deviation), third moment (skewness), forth moment (kurtosis), and best fitted distribution. TP represents exponential truncated power law, and P represents power law.

Type	*Motif id*	N_{total}	N_{tail}	N_{tail}/N_{total}	$D_{\mathrm{fit\_min}}$	D_{emp_max}	D	*p-value*	α	κ	Mean	Standard deviation	Skewness	Kurtosis	Fitted distribution
Location Motifs	20	2636765	22702	0.9%	0.4	80.64	0.037	0.904	1.47	33	3.63	6.20	3.56	16.36	TP
	34	1317551	122748	9.3%	0.4	57.27	0.039	1	0.96	13	4.61	5.91	2.31	6.39	TP
	30	274430	2692	1.0%	0.5	50.47	0.034	0.919	1.72	17	2.26	3.23	3.72	18.47	TP
	31	15155	-	-	0.6	41.59	0.027	0.892	2.46	-	1.58	2.02	4.99	37.01	P
	41	387935	81199	20.9%	0.5	60.04	0.033	1	0.70	8	4.91	5.27	1.87	4.07	TP
	43	66716	3682	5.5%	0.6	44.59	0.034	0.901	1.35	10	3.00	3.52	2.62	8.71	TP
	49	3164	-	-	0.6	20.50	0.033	0.861	1.88	-	1.99	2.33	3.31	13.52	P
	51	74435	21959	29.5%	0.5	49.05	0.029	1	0.39	6	4.79	4.57	1.66	3.27	TP
	53	9148	1683	18.4%	0.6	25.18	0.027	0.998	0.98	5	3.01	3.05	2.15	5.54	TP
Activity Motifs	12	60687	439	0.7%	0.5	61.27	0.035	0.878	1.60	27	3.09	4.88	3.73	18.83	TP
	20	2274136	108223	4.8%	0.4	79.04	0.039	0.916	1.14	19	4.65	6.68	2.80	10.36	TP
	21	1178982	12863	1.1%	0.5	80.64	0.033	0.962	1.50	24	3.38	5.09	3.43	16.60	TP
	33	791587	47201	6.0%	0.4	73.52	0.038	0.904	1.11	14	4.00	5.53	2.93	11.98	TP
	37	112554	104	0.1%	0.5	56.54	0.034	0.898	1.87	32	2.28	3.58	4.06	21.91	TP
	30	94658	2811	3.0%	0.5	44.78	0.037	0.88	1.41	14	2.92	3.90	2.87	10.22	TP
	35	73213	76	0.1%	0.6	44.29	0.030	0.895	2.08	24	1.95	2.58	4.32	26.64	TP
	311	55238	150	0.3%	0.5	58.05	0.034	0.842	1.80	28	2.38	3.77	4.48	29.06	TP
