# Supplementary material for: Characterizing preferred motif choices and distance impacts
Source: PLoS One. 2019 Apr 16;14(4):e0215242. doi: 10.1371/journal.pone.0215242 (PMC6467417; doi:10.1371/journal.pone.0215242)
Supplement: S1 Data — (ZIP) [file pone.0215242.s002.zip › README.pdf]

Characterizing preferred motif choices and distance impacts

Jinzhou Cao [a]; Qingquan Li* [a, b, c]; Wei Tu* [b, c]; Feilong Wang [d];

[a] State Key Laboratory of Information Engineering in Surveying, Mapping, and Remote Sensing, Wuhan University, Wuhan, P.R. China
[b] Shenzhen Key Laboratory of Spatial Information Smart Sensing and Services and Research Institute for Smart Cities, Department of Urban Informatics, School of Architecture and Urban Planning, Shenzhen University, Shenzhen, P.R. China
[c] Key Laboratory for Geo-Environmental Monitoring of Coastal Zone of the National Administration of Surveying, Mapping and GeoInformation, Shenzhen University, Shenzhen, P.R. China
[d] Department of Civil and Environmental Engineering, University of Washington, Seattle, USA.
* Email: tuwei@szu.edu.cn

Overview of shared data

To allow the attempt a replication of the main results of our study, we share the data. We note that due to the nature of our data use agreement of the original data provider, not all original data can be shared. The data in this release consist of the following:
1. Frequency distribution for the location-based motifs. This includes data by calculating the frequency distribution for the 197 location motif types detected in this study, as described in the manuscript. This data is used to create Figure 3(a) and to calculate the fitting results displayed in Figure 4(a), 5(a) and the correlations of frequency and average degree for location-based motifs displayed in Figure 5(c) in the manuscript and Figure S2 in the Supplementary Material.
2. Frequency distribution for the activity-based motifs. This includes data by calculating the frequency distribution for the 24 activity motif types detected in this study, as described in the manuscript. This data is used to create Figures 3(b) and to calculate the fitting results displayed in Figure 4(b), 5(b) and the correlations of frequency and average degree for activity-based motifs displayed in Figure 5(d) in the manuscript and Figure S2 in the Supplementary Material.
3. Joint frequency distribution for the top 10 location-based motifs and the top 10 activity-based motifs. This includes data by calculating the joint frequency distribution for the top 10 location motifs and the top 10 activity motifs, as described in the manuscript. This data allows the reproduction of Figure 4(c)-(d) in the manuscript.
4. Average travel Distance for the individuals with corresponding location node number, activity node number, location motif id and activity motif id. This data can be used to calculate the fitting results displayed in Figure 6-8 in the manuscript and Figure S3-S7 in the

Supplementary Material. The detailed summary of fitting results used in this data is displayed in the Table S2-S3 in the Supplementary Material.

Descriptions and file format

1. Frequency distribution for the location-based motifs.

All data in one file: `Freq_LocMotif.csv`

Format (as given by the header):

Locmotif_Id, Freq, Prop, Node

where *Locmotif_Id* refers to the id number of location-based motifs detected in this study. *Freq* refers to the frequency for each motif type. *Prop* refers to the proportion of each motif type. *Node* refers to the node number.

2. Frequency distribution for the activity-based motifs.

All data in one file: `Freq_ActiMotif.csv`

Format (as given by the header):

Actimotif_Id, Freq, Prop, Node

where *Actimotif _Id* refers to the id number of activity-based motifs detected in this study. *Freq* refers to the frequency for each motif type. *Prop* refers to the proportion of each motif type. *Node* refers to the node number.

3. Joint frequency distribution for the top 10 location-based motifs and the top 10 activity-based motifs.

All data in one file: `Freq_JointMotif_Top10.csv`

Format (as given by the header):

Locmotif_Id, Actimotif_Id, Freq, Prop

where *Locmotif_Id* refers to the id number of location-based motifs. *Actimotif _Id* refers to the id number of activity-based motifs. Freq refers to the frequency for the joint motifs. Prop refers to the proportion of the joint motifs.

4. Average travel Distance for the individuals

All data in one file: `Ave_Distance_all.csv`

Format (as given by the header):

Uid, LocNumnode, ActiNumnode, AveDis, LocmotifidW, actimotifidW

where *Uid* refers to the unique user. *LocNumnode* refers to the location node number. *ActiNumnode* refers to the activity node number. *AveDis* refers to the average travel distance with the unit of meter. *LocmotifidW* refers to the id number of location-based motifs. *ActimotifidW* refers to the id number of activity-based motifs.
